# Gut Microbiota in Colorectal Cancer: Mechanistic Insights, Clinical Strategies, and a Regional Perspective with a Focus on Sichuan, China

**DOI:** 10.3390/cancers18111693

**Published:** 2026-05-22

**Authors:** Zuoliang Liu, Mia Yang Ang, Chin Siang Kue

**Affiliations:** 1 Department of Gastrointestinal Surgery, Affiliated Hospital of North Sichuan Medical College, Maoyuan South Road, Shunqing District, Nanchong 637000, China; liuzuoliang@nsmc.edu.cn; 2 School of Graduate Studies, Post Graduate Centre, Management and Science University, Seksyen 13, Shah Alam 40100, Selangor, Malaysia; 3 Institute of Hepatobiliary Pancreatic Intestinal Diseases, North Sichuan Medical College, Maoyuan South Road, Shunqing District, Nanchong 637000, China; 4 National Clinical Key Specialty (General Surgery), Sub-Center of National Clinical Research Center for Digestive Diseases, Sichuan Clinical Research Center for Digestive Diseases, Maoyuan South Road, Shunqing District, Nanchong 637000, China; 5 Department of Diagnostic and Allied Health Science, Faculty of Health and Life Sciences, Management and Science University (MSU), University Drive, Off Persiaran Olahraga, Section 13, Shah Alam 40100, Selangor, Malaysia

**Keywords:** colorectal cancer, gut microbiota, microbial dysbiosis, carcinogenesis, microbial metabolites, epigenetic regulation, DNA methylation, immune regulation, Sichuan, China, regional diet, fecal microbiota transplantation, precision medicine

## Abstract

CRC remains a major cause of cancer-related morbidity and mortality worldwide. In recent years, the gut microbiota has gained increasing attention in CRC research. Intestinal microbes are not passive bystanders in tumor development. They may promote persistent inflammation, disrupt epithelial barrier integrity, alter microbial metabolites, and affect host immune and signaling pathways. Emerging evidence also suggests that microbiota-related metabolites and microbial functional alterations may influence host epigenetic regulation, including DNA methylation and chromatin-associated signaling, thereby further shaping colorectal carcinogenesis. Together, these changes can create a microenvironment that favors tumor initiation and progression. Several bacterial species, including *Fusobacterium nucleatum*, *Parvimonas micra*, and *Peptostreptococcus anaerobius*, have been repeatedly associated with CRC. In contrast, beneficial commensal microbes and their metabolites, especially short-chain fatty acids, may help maintain intestinal homeostasis and limit tumor-promoting processes. Because the gut microbiota is strongly shaped by diet, lifestyle, and environmental exposure, regional differences are also relevant. This is particularly important in Sichuan, China, where distinctive dietary habits and environmental features may influence microbial patterns associated with CRC risk and disease behavior. This review summarizes the main mechanisms linking the gut microbiota to CRC, examines the regional context of Sichuan, China, and discusses current and emerging clinical strategies. These include dietary intervention, probiotics, fecal microbiota transplantation, and microbiome-informed approaches to prevention, diagnosis, and treatment.

## Simple Summary

Colorectal cancer (CRC) is one of the most common cancers worldwide. It develops through interactions between tumor cells and the intestinal environment. Among the factors involved, the gut microbiota has received growing attention. Changes in gut microbes may affect inflammation, metabolism, immune function, and barrier integrity, which can support tumor development. At the same time, beneficial microbes may help maintain intestinal health. Because the gut microbiota is shaped by diet, lifestyle, and environmental exposure, regional context also matters. This is especially relevant in Sichuan, China, where distinctive dietary and environmental features may influence microbial patterns related to colorectal cancer. Better understanding of these interactions may support improved prevention, earlier detection, and more personalized management.

## 1. Introduction

Colorectal cancer (CRC) poses a formidable global health burden, with 1.9 million new cases and 904,019 deaths annually, ranking as the second leading cause of cancer-related mortality [1]. While genetic predisposition contributes to CRC, the gut microbiota has emerged as a critical mediator of carcinogenesis, influencing immune surveillance, metabolic homeostasis, and cellular signaling [2].

The human gut hosts a dynamic consortium of bacteria, viruses, fungi, and archaea that collectively sustain digestive health, immunity, and metabolism [3]. In homeostasis, this ecosystem fosters resilience through beneficial metabolite production, pathogen inhibition, and immune modulation. Conversely, the onset of CRC has been strongly correlated with dysbiosis, a condition marked by diminished microbial diversity and an enrichment of pathogenic taxa [4].

Geographic disparities in CRC incidence reflect intricate interactions between genetics, environment, and lifestyle, particularly diet. Westernized diets are often rich in processed foods and poor in fiber. Such patterns have been associated with a higher risk of CRC. By contrast, traditional plant-based diets are generally associated with a lower risk [5]. These dietary patterns sculpt distinct microbial signatures that either mitigate or exacerbate cancer risk. Notably, while CRC rates have declined in certain regions, other areas have experienced a rapid increase, often paralleling shifts toward processed food diets and lifestyle changes [6].

Beyond diet, the gut microbiota actively participates in vital physiological processes: fermenting indigestible fiber, preserving epithelial barrier function, synthesizing vitamins, and fine-tuning immunity [7]. Dysbiosis disrupts these functions, fueling chronic inflammation, impairing immune defenses, and generating genotoxic metabolites that collectively drive tumorigenesis [8].

Understanding microbiota–CRC interactions requires attention not only to molecular mechanisms but also to ecological and regional factors. Many previous reviews have summarized the general link between gut dysbiosis and colorectal cancer. However, these aspects are often addressed separately. The present review seeks to bring them together. In addition to established mechanistic pathways, it discusses epigenetic regulation, microbial community and network effects, clinical and translational strategies, and the influence of regional diet and environment. Particular attention is given to Sichuan, China, as a regionally relevant and hypothesis-generating example. This approach aims to provide a more contextualized and clinically relevant view of microbiota–CRC interactions.

## 2. Gut Microbiota Composition and Function

The human gut microbiota begins to establish at birth. Its early composition is shaped by several factors. These include the mode of delivery, such as vaginal birth or cesarean section [9,10]. Feeding practices, including breastfeeding and formula feeding, also influence early microbial colonization [11,12,13]. Early environmental exposures further contribute to this process [14,15]. Vaginal delivery (VD) and breastfeeding promote maternal-like microbial communities, while caesarean section (CS) and formula feeding are associated with distinct patterns of microbial colonization [16]. Dominguez-Bello et al. performed one of the earliest high-throughput microbiome studies on this topic. They found that the gut microbiota of VD infants resembled their mothers’ vaginal microbiota. The dominant taxa included *Lactobacillus*, *Prevotella*, and *Sneathia* spp. By contrast, C-section newborns harbored bacterial communities more similar to skin microbiota. These communities were dominated by *Staphylococcus*, *Corynebacterium*, and *Propionibacterium* spp. [17]. Subsequent work leveraging more advanced methodologies has further elucidated these dynamics. For instance, Hanachi et al. used high-resolution shotgun sequencing to analyze gut microbiota in Tunisian newborns delivered by CS or VD over the first month (Day 0, Day 15, and Day 30). Interestingly, both groups showed similar bacterial diversity, with early shifts in microbial composition. However, CS infants had reduced *Bacteroides* and increased ESKAPE pathogens after two weeks, despite overall similarities in dominant phyla [10]. Beyond delivery mode, infant feeding type also plays a crucial role in shaping early gut microbial development. Inchingolo et al. [13] conducted a systematic review comparing the gut microbiota of breastfed and formula-fed infants, highlighting that breastfeeding is associated with greater microbial diversity and higher levels of beneficial bacteria such as *Bifidobacterium* and *Lactobacillus*. In contrast, formula feeding correlated with increased abundance of potentially pathogenic taxa like *Clostridium difficile* and *Enterobacteriaceae*. The infant gut microbiota remains dynamic during early life, gradually maturing into an adult-like composition by the age of 2–3. Although 60–70% of the adult microbiota remains relatively stable, the remainder adapts to dietary, lifestyle, and physiological changes [18,19]. Microbial diversity peaks in adulthood but continues to fluctuate with age, disease, or environmental shifts. These early-life factors are not presented here as direct causes of colorectal cancer. However, they may contribute to long-term differences in microbial composition and may therefore form part of the background variability seen in adult microbiome cohorts.

**Figure 1:**
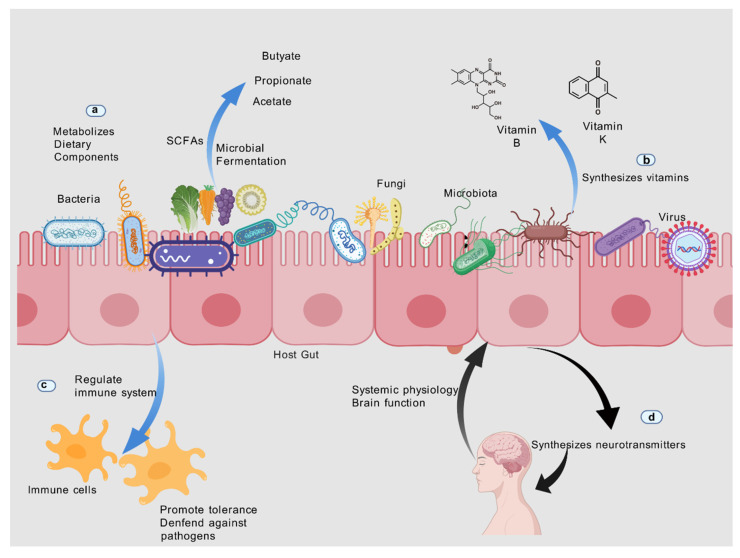
Physiology functions of the gut microbiota (Created with BioGDP.com). The gut microbiota supports host homeostasis through several interconnected functions, including fermentation of indigestible dietary substrates, production of SCFAs, regulation of mucosal immunity, maintenance of epithelial barrier integrity, and synthesis of essential vitamins and bioactive compounds. These activities collectively contribute to metabolic balance, immune tolerance, and protection against pathogen colonization. SCFAs: short-chain fatty acids.

The gut microbiota supports host health through three primary functions (Figure 1) [20]: (i) it metabolizes indigestible dietary components, producing short-chain fatty acids (SCFAs) like butyrate, propionate, and acetate, which maintain gut barrier integrity and regulate metabolism [21];. (ii) it educates and regulates the immune system, promoting tolerance [22] while defending against pathogens through competitive exclusion, antimicrobial production, and mucosal reinforcement [23] (iii) it synthesizes essential vitamins (B12, K) and neurotransmitters, influencing systemic physiology and brain function [24]. These functions collectively reveal the microbiota’s central role in maintaining host homeostasis.

## 3. Molecular Mechanisms of Microbiota–CRC Interactions

### 3.1. Dysbiosis and Tumorigenesis

CRC-associated dysbiosis is characterized by specific changes in gut microbial composition that actively contribute to tumorigenesis [25]. This microbial imbalance usually involves a loss of overall microbial diversity. It is often accompanied by an expansion of pathogenic bacterial species. These microbes can induce inflammation and contribute to cellular transformation. In this context, Coker et al. [26] revealed significant alterations in gut bacterial composition across CRC stages. Species such as *Fusobacterium nucleatum*, *Peptostreptococcus anaerobius*, and *Parvimonas micra* were enriched in CRC, while beneficial taxa like *Roseburia* and *Eubacterium* were depleted. These microbial signatures showed high diagnostic accuracy in distinguishing CRC, adenoma, and healthy controls, with an AUC greater than 0.90. This finding suggests that they may serve as non-invasive biomarkers for early CRC detection. Table 1 summarizes pathogenic bacteria species that promote tumorigenesis and protective species that inhibit tumor formation in CRC, and their mechanisms. Among these microbes, *Fusobacterium nucleatum* has emerged as a key oncogenic bacterium that enhances tumor progression through multiple mechanisms. One of the surface adhesins of *F. nucleatum*, Rad D, mediates interactions with various Gram-positive bacteria and fungi [27,28]. These interactions help promote biofilm formation. Its lipopolysaccharides can also activate NF-κB signaling. As a result, pro-inflammatory cytokines such as TNF-α, IL-6, and IL-1β are released [29,30]. These inflammatory mediators create a tumor-promoting microenvironment that supports immune evasion and malignant transformation.

Other pathogenic species contribute to CRC development through distinct mechanisms. Enterotoxigenic *Bacteroides fragilis* produces the Bft toxin, which induces DNA double-strand breaks and aberrantly activates the Wnt/β-catenin pathway, driving uncontrolled epithelial proliferation [39,40]. Similarly, genotoxic strains of *Escherichia coli* harboring the polyketide synthase (pks) genomic island produce colibactin, a potent genotoxin that causes DNA damage in intestinal epithelial cells [34]. *Enterococcus faecalis* further contributes to genomic instability through the generation of reactive oxygen species (ROS), which induce oxidative stress and DNA mutations in colonocytes [41].

**Figure 2:**
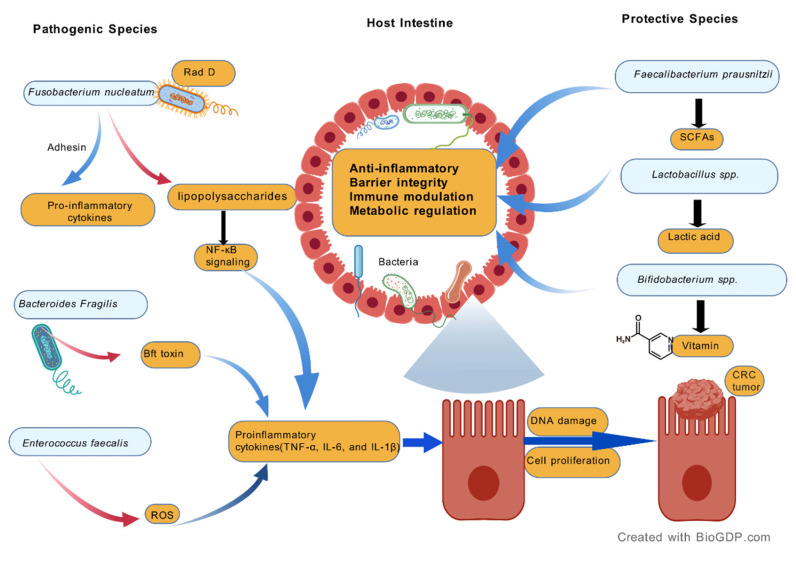
Mechanistic interactions between gut microbiota and CRC progression. CRC-associated dysbiosis may promote tumor initiation and progression through multiple interacting mechanisms. These include epithelial barrier disruption, chronic inflammation, microbial toxin production, oxidative stress, DNA damage, and altered metabolite profiles. At the same time, depletion of beneficial commensals and reduced SCFA production may weaken mucosal protection and anti-inflammatory signaling, thereby favoring a pro-carcinogenic intestinal environment. ROS: reactive oxygen species; CRC: colorectal cancer; DNA: deoxyribonucleic Acid; SCFAs: short-chain fatty acids; NF-κB: Nuclear Factor kappa-light-chain-enhancer of activated B cells.

In contrast, protective bacterial species are frequently depleted in CRC patients. Beneficial microbes such as *Faecalibacterium prausnitzii*, *Lactobacillus* species, and *Akkermansia muciniphila* normally maintain gut homeostasis by strengthening epithelial barrier function and producing anti-inflammatory metabolites [35]. Butyrate-producing Firmicutes are particularly important in gut homeostasis. When these bacteria are reduced, the availability of short-chain fatty acids (SCFAs) also declines [42]. SCFAs are essential for maintaining colonocyte health and limiting inflammation. At the same time, pathogenic species may expand. Together, these changes create a permissive environment for tumor initiation and progression (Figure 2) [20].

CRC-associated dysbiosis should not be understood only as the presence of single pathogenic bacteria. Increasing evidence suggests that microbial communities, co-occurrence patterns, and ecological succession are also important [43]. Taxa such as *Fusobacterium nucleatum*, *Parvimonas micra*, and *Peptostreptococcus* spp. may act within a broader bacterial network rather than in isolation. This may help explain why CRC-associated taxa are detected inconsistently across studies. It is also consistent with the driver–passenger model [44], in which early bacteria disturb the mucosal environment and later bacteria colonize the altered tumor niche. From this perspective, CRC may reflect not only pathogen enrichment but also loss of protective microbial networks, especially butyrate-producing commensals [45].

### 3.2. Inflammatory Pathways and Immune Modulation

**Figure 3:**
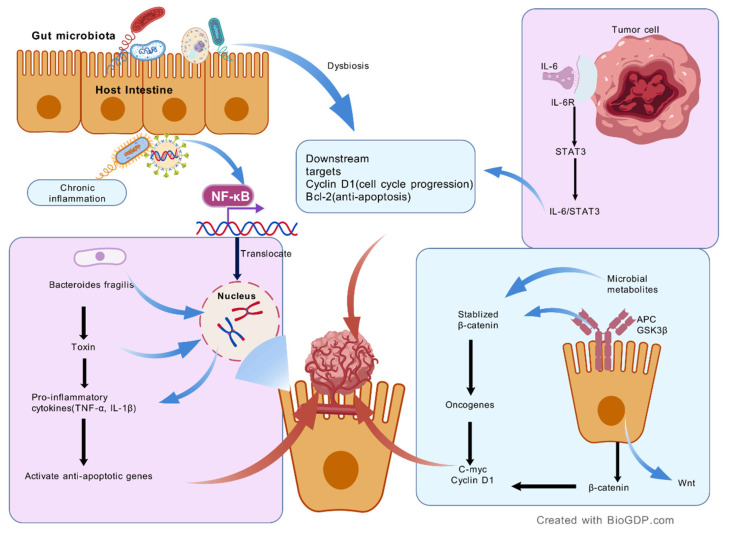
Host–microbe interactions in colorectal cancer: Roles in inflammation, immune modulation, and oncogenic signaling. This schematic summarizes how microbial dysbiosis and microbial products may influence host signaling pathways involved in colorectal carcinogenesis. Key pathways include NF-κB-mediated inflammatory activation, IL-6/STAT3-driven tumor-promoting signaling, and disruption of the APC/GSK3β/β-catenin regulatory axis. Together, these interactions may enhance epithelial proliferation, immune evasion, and tumor progression. IL-6, interleukin-6; APC, adenomatous polyposis coli; STAT3, Signal Transducer and Activator of Transcription 3; GSK3β, glycogen synthase kinase 3 beta.

The gut microbiota drives CRC progression through interconnected inflammatory and immune-modulatory pathways (Figure 3) [20]. Chronic inflammation induced by pathogenic bacteria can activate nuclear factor-κB (NF-κB) signaling. This pathway upregulates pro-inflammatory cytokines, such as TNF-α and IL-1β. It also increases the expression of anti-apoptotic genes. Microbial ligands and oxidative stress can further stimulate NF-κB activation. Once activated, NF-κB translocates to the nucleus. It then initiates the transcription of genes that sustain inflammation and promote tumorigenesis [46].

The interleukin-6 (IL-6)/STAT3 axis further amplifies oncogenic signaling. Elevated IL-6 in CRC binds to its receptor, activating STAT3 to promote tumor cell survival and proliferation [47]. Downstream targets include cyclin D1 (cell cycle progression) and Bcl-2 (anti-apoptosis), fostering a pro-tumorigenic microenvironment.

Dysbiosis also disrupts the Wnt/β-catenin pathway, a pivotal regulator of intestinal epithelial proliferation [48]. In healthy states, β-catenin is degraded by the APC (adenomatous polyposis coli)/GSK3β destruction complex. However, microbial metabolites or APC mutations stabilize β-catenin, activating oncogenes (e.g., c-myc, cyclin D1). Pathogens like *Bacteroides fragilis* exacerbate this via toxin-mediated Wnt activation.

### 3.3. Metabolic Interactions and Genotoxicity

The gut microbiota profoundly influences CRC development through its metabolic output, generating both protective and pathogenic compounds that shape intestinal homeostasis. SCFAs, particularly butyrate, propionate, and acetate, represent key beneficial metabolites with multiple anti-inflammatory and anti-tumorigenic properties [49] (Table 2). Butyrate, the most biologically active SCFAs, serves as the primary energy source for colonocytes and exerts potent anti-cancer effects through: (1) histone deacetylase (HDAC) inhibition that modulates inflammatory gene expression, (2) promotion of epithelial barrier integrity, (3) induction of apoptosis in transformed cells, and (4) cell cycle regulation via p21/p27 activation and CDK inhibition. Propionate enhances regulatory T-cell activity [50] and gluconeogenesis regulation, whereas acetate, the most abundant SCFAs, maintains luminal acidity at a level unfavorable for pathogens and supports lipid metabolism [51]. Additionally, commensal bacteria synthesize essential micronutrients including vitamin K2 and B vitamins (B12 and folate) that support DNA stability and repair mechanisms [52].

Conversely, dysbiosis leads to the accumulation of genotoxic metabolites that drive carcinogenesis through distinct mechanisms. For instance, secondary bile acids (deoxycholic acid and lithocholic acid), produced by Clostridium and Eubacterium species, induce DNA damage, disrupt barrier function, and activate pro-inflammatory NF-κB signaling. Protein fermentation can also generate several harmful compounds. Ammonia produced by *Bacteroides* and *Clostridium* species may cause direct epithelial toxicity. Hydrogen sulfide, particularly that associated with *Desulfovibrio*, has direct genotoxic potential. p-Cresol may further contribute to oxidative DNA damage. Elevated polyamines (putrescine and spermidine) from proteolytic fermentation further stimulate excessive cellular proliferation when dysregulated.

The equilibrium between these opposing metabolic forces determines CRC risk. A balanced microbiota helps create a protective intestinal environment. This effect is partly mediated by SCFAs, which have anti-inflammatory properties and support genomic stability. In contrast, dysbiosis promotes a pro-carcinogenic environment. This state is characterized by chronic inflammation, barrier dysfunction, and cumulative DNA damage. This metabolic duality highlights the microbiota’s central role in colorectal carcinogenesis and presents promising targets for therapeutic intervention.

### 3.4. Epigenetic Mechanisms Linking Diet, Microbiota, and Colorectal Cancer

Epigenetic regulation represents an important mechanistic interface linking diet, the gut microbiota, and colorectal carcinogenesis. In CRC, aberrant DNA methylation is a well-recognized hallmark that contributes to the silencing of tumor suppressor genes and the dysregulation of oncogenic pathways [63,64]. Increasing evidence suggests that gut microbes may influence these epigenetic processes not only indirectly through inflammation and metabolic stress but also more directly through alterations in microbial functions involved in one-carbon metabolism and methyl-donor availability [65].

In addition to DNA methylation, microbiota-derived metabolites may modulate histone and chromatin states. Short-chain fatty acids, particularly butyrate, are known to inhibit histone deacetylases and thereby regulate transcriptional programs involved in cell cycle arrest, apoptosis, barrier integrity, and immune homeostasis. Other microbial metabolites may further influence epigenetic remodeling by affecting folate-, methionine-, and S-adenosyl-L-methionine-related pathways [66]. Thus, the diet–microbiota axis may shape CRC risk not only through inflammation and metabolite imbalance, but also through epigenetic reprogramming of intestinal epithelial and immune cells. This perspective broadens the mechanistic framework of microbiota–CRC interactions and highlights epigenetic regulation as a promising area for biomarker development and therapeutic intervention.

Importantly, these molecular and epigenetic mechanisms should not be viewed in isolation from the regional dietary and environmental context. Diet-specific exposures may shape microbial composition and metabolite output in ways that converge with the oncogenic processes discussed above. In the context of Sichuan, this may be particularly relevant for preserved, smoked, and processed meat products, which may increase exposure to N-nitroso compounds, heme-related oxidation products, and other potentially carcinogenic intermediates. Such exposures may contribute to DNA damage, epithelial barrier disruption, oxidative stress, and pro-inflammatory signaling while also potentially interacting with broader metabolic and epigenetic regulatory pathways involved in colorectal carcinogenesis. At the same time, fiber-rich and fermented dietary components may exert partially opposing effects by supporting beneficial microbes, short-chain fatty acid production, and microbial functions associated with intestinal homeostasis. Taken together, these interactions provide an important conceptual bridge between the mechanistic framework outlined above and the region-specific dietary patterns discussed below.

## 4. Regional Perspectives: A Focus on Sichuan, China

### 4.1. Dietary Influences on Microbiota Composition and CRC Risk

The dietary patterns of Sichuan province provide a useful regional perspective on the relationship between diet, gut microbiota, and CRC risk. Sichuan cuisine is characterized by a distinctive combination of spicy, fermented, and preserved foods, which may influence gut microbiota composition and CRC risk in different ways. On the one hand, traditional dietary components such as chili peppers, fermented vegetables, and fiber-containing plant foods may support microbial diversity and promote the growth of beneficial bacteria [67]. Certain fermented foods may also introduce live microorganisms with potential probiotic properties- [68,69]. These effects may contribute to short-chain fatty acid (SCFA) production, maintenance of intestinal barrier integrity, and modulation of local immune responses. Similarly, the bioactive compounds in Sichuan peppercorns exhibit targeted antimicrobial activity against enteric pathogens, yet preserve commensal species.

Fermented foods, such as pickled vegetables and fermented bean pastes including doubanjiang, are central to the traditional Sichuan diet [70]. They may benefit gut health in several ways. They can introduce live microorganisms with potential probiotic properties. They may also enhance microbial diversity, compete with pathogens for ecological niches, and generate metabolites with immunomodulatory effects. Regular consumption of these foods may help sustain beneficial taxa, particularly the butyrate-producing *Faecalibacterium prausnitzii* and the mucin-degrading *Akkermansia muciniphila*. Both have been linked to improved metabolic health and a lower risk of CRC. The high fiber content of the traditional Sichuan diet may provide additional support for the growth of these protective microbes.

By contrast, some foods that are commonly consumed in this region may increase CRC risk. Processed and preserved meats, including Sichuan sausage, smoked bacon, and spiced duck, may disturb gut microbial balance. This shift may favor potentially harmful taxa such as *Bacteroides* and *Clostridium* species, while reducing beneficial bacteria. It may also increase the production of carcinogenic metabolites, including secondary bile acids and N-nitroso compounds, which have been implicated in colorectal tumorigenesis [57]. In addition, high-salt preserved foods may weaken mucosal barrier integrity and intensify pro-inflammatory changes in the intestinal microenvironment. Diet-related carcinogenic effects may also depend on microbial consortia rather than on a single organism. Under high-fat or meat-rich dietary conditions, bacterial networks may promote the conversion of primary bile acids into carcinogenic secondary bile acids such as deoxycholic acid [71]. Processed and smoked meats may also increase exposure to nitrosamine precursors and fecal N-nitroso compounds [72]. This network-based view may help explain why associations between individual bacteria and CRC are not always reproduced across geographic cohorts.

This regional perspective is supported by broader epidemiological evidence from China. However, direct Sichuan-specific microbiome–CRC studies remain scarce. Most references cited in this section provide general dietary, microbiological, or epidemiological background rather than direct microbiome data from Sichuan. National and subnational studies have shown clear geographic variation in CRC burden across China [73]. Western and southwestern regions have experienced notable changes in incidence and mortality. These findings highlight the importance of considering dietary transition, food preservation practices, and local environmental factors when discussing CRC risk in Sichuan. Local dietary exposures may also shape stool microbial and metabolite profiles independently of disease status. For this reason, fermented foods, preserved vegetables, and smoked meat products should be regarded as important contextual variables when interpreting regional microbial signals.

Currently available evidence does not support a simplistic interpretation of traditional Sichuan dietary exposure as uniformly protective or harmful. Fermented foods, pickled vegetables, processed meats, and broader westernized dietary elements may have different effects. These effects may be related to changes in microbial composition, metabolite production, and mucosal inflammation [74]. Therefore, the Sichuan-focused discussion should be viewed as a biologically plausible and hypothesis-generating regional framework rather than a definitive causal model.

Overall, the effect of Sichuan dietary patterns on CRC risk is likely determined by the balance between protective and harmful dietary components. While plant-based, fermented, and fiber-rich foods may support a more favorable microbial ecosystem, excessive consumption of processed, preserved, and high-salt foods may shift the gut microbiota toward a pro-carcinogenic state. Therefore, the net effect of Sichuan dietary habits on colorectal health is likely determined by the overall dietary balance, frequency of exposure, and interactions with host and microbial factors. Further population-based studies integrating dietary assessment, microbiome profiling, and CRC outcomes in Sichuan are still needed to validate these region-specific hypotheses [73,74].

### 4.2. Environmental and Genetic Determinants of Microbiota and CRC Risk in Sichuan

The gut microbiota in Sichuan populations is shaped by a dynamic interplay between environmental pressures and host genetics, creating a unique ecological landscape with significant implications for CRC risk. Rapid industrialization has introduced environmental stressors, particularly airborne PM2.5 and waterborne heavy metals (lead, cadmium, and arsenic), which disrupt microbial communities through distinct yet interconnected mechanisms. PM2.5 particles translocate to the gut lumen, where they drive a pro-inflammatory shift characterized by Proteobacteria expansion and depletion of beneficial taxa (*Faecalibacterium* and *Bifidobacterium*). Exposure to contaminated water sources may further worsen dysbiosis. It can favor the growth of metal-resistant pathobionts. It may also disturb the balance between Firmicutes and Bacteroidetes. This type of imbalance is commonly associated with CRC-prone microbiomes. Microbial profiles in urban centers in Sichuan are becoming more similar to those seen in Westernized populations. This shift has been associated with increased CRC susceptibility. It also suggests that environmental degradation may strongly affect the stability of the gut ecosystem [75].

Antibiotic exposure is another important non-dietary factor that can alter the gut microbiota. Its effects are not limited to broad-spectrum agents. Even narrower-spectrum antibiotics may disrupt microbial diversity, composition, and metabolic function. An updated meta-analysis reported that antibiotic use was associated with increased risk of colorectal neoplasia. Stronger signals were seen for some classes, including penicillins, cephalosporins, and anti-anaerobic antibiotics [76]. A Swedish nationwide study also found that antibiotic use was associated with a higher risk of proximal colon cancer. The same study suggested site-specific and sex-related differences [77]. In CRC research, this is important because prior antibiotic exposure may affect adult microbiome structure independently of diet. It may therefore contribute to inter-individual and cohort-level heterogeneity in microbiome studies. This issue may also be relevant to the broader discussion of early-onset colorectal cancer [78].

At the genetic level, Sichuan populations possess distinct variants in immune regulatory pathways (Human Leukocyte Antigen, HLA and Toll-like receptors, TLR) and epithelial integrity genes (tight junction proteins) that modulate host-microbe crosstalk [79]. These polymorphisms influence microbial community resilience, with certain variants conferring protection against environmental insults, whereas others amplify susceptibility to dysbiosis. For instance, TLR variants may predispose individuals to pollution-induced microbial shifts, whereas protective HLA alleles could maintain homeostasis despite environmental stressors. Critically, these genetic factors do not operate in isolation but interact dynamically with environmental exposures, creating population-specific patterns of CRC risk.

This gene–environment–microbiota triad highlights the need for precision prevention strategies in Sichuan. A better understanding of how regional pollution interacts with local genetic factors may help explain changes in gut microbial ecology. It may also help identify populations at higher risk of CRC. On this basis, more targeted interventions could be developed. These may range from pollution mitigation to personalized microbiota modulation. Such approaches may be particularly relevant in a rapidly developing region like Sichuan.

### 4.3. Comparative Analysis of Global Dietary Structures

Western Populations: Western diets are high in fat and processed foods but low in fiber, promoting gut microbiota dysbiosis characterized by Bacteroides dominance, reduced microbial diversity, and diminished SCFA production [80]. This profile correlates with elevated CRC incidence in North America and Europe, mediated through impaired gut barrier function, chronic inflammation, and the generation of harmful metabolites like trimethylamine-N-oxide (TMAO) from red meat processing (Table 3) [81,82]. Supporting the link between diet, microbiota, and CRC, Yang et al. provided compelling evidence in murine models. Their study showed that a high-fat diet (HFD) promoted colorectal tumorigenesis. This effect was associated with gut microbial dysbiosis. The dysbiotic pattern was characterized by enrichment of pathogenic *Alistipes* species and depletion of the probiotic *Parabacteroides distasonis* [83]. HFD also leads to metabolomic alterations, notably elevated levels of lysophosphatidic acid (LPA), which enhances epithelial proliferation and compromises barrier integrity. Fecal microbiota transplantation from HFD-fed donors recapitulated these oncogenic effects in germ-free mice. This finding highlights the critical role of HFD-modulated microbiota in colorectal cancer pathogenesis. It also suggests an important contribution of microbiota-derived metabolites to this process. The diet–microbiota axis emerges as a key modifiable risk factor, with low fiber intake reducing protective taxa (Faecalibacterium and Roseburia), thereby promoting pro-inflammatory pathways, a pattern further linked to metabolic disorders and inflammatory bowel diseases prevalent in Western populations.

Traditional Asian Diets: Traditional diets in East Asian countries such as Japan and Korea are rich in fermented foods, including miso, natto, and kimchi, as well as seaweed, seafood, and fermented soybean products. These dietary patterns promote a specialized gut microbiota featuring high abundances of *Bifidobacterium* and *Lactobacillus*, along with unique adaptations like *Bacteroides plebeius*, which encodes enzymes for digesting seaweed polysaccharides [79,80]. Reflecting the functional impact of such dietary patterns, a recent study [81] demonstrates that *B. plebeius* colonization, promoted by a seaweed-supplemented diet, significantly suppresses colitis-associated colon cancer in murine models. This protective effect was associated with changes in the gut microbiota. The abundance of pro-tumorigenic bacteria was reduced. At the same time, the production of anti-inflammatory and tumor-suppressive metabolites increased. These included propionic acid and ursodeoxycholic acid. Transcriptomic profiling further revealed downregulation of inflammatory and oncogenic gene expression. Notably, *B. plebeius* exerted tumor-inhibitory effects even without dietary seaweed in germ-free mice, underscoring its therapeutic potential in modulating host-microbe interactions.

In contrast, South Asian diets-typified by Indian, Pakistani, and Bangladeshi cuisines-are traditionally plant-based and high in legumes, whole grains, spices (e.g., turmeric), and ghee, with moderate dairy consumption and limited intake of fermented foods. These diets are rich in dietary fiber and polyphenols. They promote gut microbial diversity and favor saccharolytic fermentation. This process enhances SCFA production, particularly butyrate. Butyrate is an important metabolite with anti-inflammatory and anti-carcinogenic effects.

Although both East and South Asian dietary traditions emphasize plant-derived foods, their core components differ significantly. South Asian diets focus on fiber, legumes, and bioactive spices, while East Asian diets emphasize fermentation and marine-based ingredients. These distinctions contribute to region-specific gut microbiota compositions and may underlie differing susceptibilities to chronic diseases such as colorectal cancer (CRC), obesity, and cardiovascular disease [82].

Taken together, these findings suggest that the diet-driven microbial profiles in East Asian populations—characterized by enhanced fermentation capacity, anti-inflammatory potential, and the presence of functionally adapted species such as *Bacteroides plebeius*—may contribute to the historically lower incidence of CRC and metabolic disorders in countries like Japan and Korea. The incidence of CRC in East Asia presents a complex contrast to that observed in Western countries. Although the overall CRC incidence in East Asia (25.9 per 100,000) is slightly lower than in Europe (30.4 per 100,000) and Oceania (29.8 per 100,000), substantial regional variation exists. For instance, Japan reports notably higher rates (38.5 per 100,000), approaching those of Western nations, while China and South Korea exhibit moderately lower rates. Despite the comparatively lower incidence, East Asia is witnessing a marked increase in CRC cases, particularly among younger adults-a trend less evident in many Western regions where incidence is stabilizing or declining. This divergence is likely driven by rapid urbanization, lifestyle changes, and increased exposure to metabolic and dietary risk factors. The distinct microbial landscape in East Asia—shaped by long-standing dietary practices such as high consumption of fermented foods and seaweed—may help explain Japan’s relatively low rates of CRC, obesity, and cardiovascular disease [83]. Notably, the presence of seaweed-adapted species like *B. plebeius* illustrates how traditional diets can influence microbial evolution and gut function. Together, these elements highlight the role of region-specific diets in fostering protective microbial ecosystems.

Rural African Populations: Rural African communities, with their traditional plant-based, high-fiber diets, contain gut microbiomes of exceptional diversity dominated by *Prevotella* species, a profile linked to enhanced carbohydrate fermentation and remarkably low CRC incidence despite limited healthcare access [85]. These populations, consuming minimal processed foods or animal products, maintain microbial ecosystems enriched in fiber-degrading taxa that promote metabolic resilience and anti-inflammatory environments. However, urbanization in Africa is driving dietary shifts from traditional high-fiber diets to Western-style diets, leading to changes in gut microbiota composition and metabolic profiles associated with increased CRC risk [86,87]. A cross-sectional study [88] of rural and urban South African Xhosa individuals revealed that urbanized dietary patterns were associated with reduced gut microbial diversity, enrichment of CRC-associated taxa such as *Bacteroides* and *Fusobacterium*, increased levels of fecal deoxycholic acid, and distinct food- and skin-associated microbiota. Notably, despite these differences, both groups exhibited comparable levels of SCFAs. These findings highlight the profound impact of modernization on the gut ecosystem and underscore the utility of the South African model in elucidating the global rise of CRC and the susceptibility of indigenous microbiomes to dietary Westernization.

Mediterranean Populations: The Mediterranean diet, which is rich in plant foods, olive oil, fish, and moderate red wine, shapes a gut microbiota enriched in *Prevotella* and *Faecalibacterium prausnitzii*, which enhance anti-inflammatory responses and SCFA production [84]. These microbial shifts underlie the diet’s protective effects against cardiometabolic diseases. Mediterranean dietary patterns are characterized by high intakes of polyphenols and unsaturated fats. These components help promote gut microbial diversity. Despite their distinct nutritional frameworks, these dietary patterns may converge on similar anti-inflammatory outcomes. One possible mechanism is the enhancement of *F. prausnitzii*-associated SCFA production.

Taken together, these mechanistic and regional observations indicate that CRC-associated dysbiosis should be viewed not merely as a taxonomic imbalance, but as a broader translational framework linking microbial alterations with molecular mechanisms, candidate biomarkers, and therapeutic opportunities. Across different dietary and geographic settings, gut microbial disturbances may converge on common pathogenic processes, including chronic inflammation, epithelial barrier disruption, immune dysregulation, genotoxic stress, and metabolic reprogramming. To integrate these dimensions, Table 4 summarizes the representative dysbiosis patterns implicated in colorectal cancer (CRC), together with key microbial signatures, underlying mechanisms, candidate biomarkers, intervention opportunities, and current evidence levels.

## 5. Therapeutic and Preventive Strategies

As summarized in Table 4, CRC-associated dysbiosis has both mechanistic and translational significance, encompassing microbial compositional shifts, metabolite alterations, biomarker potential, and intervention targets. These insights provide the basis for current and emerging microbiota-targeted preventive and therapeutic strategies. The main approaches under investigation in CRC include dietary modulation, probiotics, prebiotics, synbiotics, postbiotics, fecal microbiota transplantation, and other personalized interventions aimed at restoring microbial and metabolic homeostasis. These strategies, together with their proposed mechanisms, potential advantages, and current limitations, are summarized in Table 5.

### 5.1. Microbiota-Targeted Interventions

Microbiota-targeted interventions represent a promising approach to improving health by modulating the gut microbiota, which plays essential roles in digestion, immunity, and metabolism. These strategies aim to restore dysbiotic microbiota or enhance beneficial aspects of healthy microbial communities, gaining rapid interest in medical research and clinical practice. Key strategies include the use of probiotics, which introduce beneficial live bacteria to rebalance gut flora; prebiotics, which provide nutrients that selectively promote the growth of helpful microbes; and synbiotics, which combine both probiotics and prebiotics for synergistic effects. Additionally, fecal microbiota transplantation (FMT) offers a more comprehensive method by transferring a healthy donor’s stool to repopulate the gut with a diverse microbial ecosystem. Postbiotics, which consist of microbial metabolites like SCFAs, also help regulate gut and immune functions without introducing live organisms.

### 5.2. Dietary Modifications

Diet represents the most potent modulator of gut microbiota, with specific foods and dietary patterns either promoting balanced, diverse microbiomes or contributing to dysbiosis and disease.

Microbial Supplementation: Probiotics such as *Lactobacillus rhamnosus*, *B. breve*, *L. acidophilus*, *Bifidobacterium longum*, and *Saccharomyces boulardii* may help rebalance the gut microbiome. They may also enhance immune function and promote SCFA production. As a result, these probiotics may provide benefits in the management of inflammatory bowel disease, diarrhea, allergies, and antibiotic-associated disturbances. They also show promise in colorectal cancer prevention and metabolic disorder management [100].

Supporting these beneficial microbes (e.g., *Bifidobacterium* and *Lactobacillus*) are prebiotics—non-digestible fibers like fructooligosaccharides, inulin, galactooligosaccharides, and resistant starch found in foods such as garlic, bananas, onions, and whole grains [101]. These compounds nourish beneficial bacteria, stimulating their growth and activity, which in turn enhances gut barrier function, modulates immunity, and reduces inflammation. Prebiotic supplementation has shown potential in treating irritable bowel syndrome, inflammatory bowel disease, and obesity. Synbiotics combine probiotics and prebiotics to create a synergistic effect, improving microbial stability and enhancing overall gut health more effectively than either component alone [102]. They help alleviate symptoms of IBS, IBD, and diarrhea, support immune function, and are being explored for their role in managing obesity and type 2 diabetes by improving glucose metabolism and reducing systemic inflammation.

Dietary Pattern Interventions: Diets rich in dietary fiber and polyphenols represent important interventions for maintaining gut microbial diversity and promoting the growth of beneficial microorganisms. Dietary fiber can be classified into soluble fiber, insoluble fiber, and resistant starch. Soluble fiber is found in fruits, oats, barley, citrus, and legumes. Insoluble fiber is abundant in nuts, whole grains, and vegetables. Resistant starch is present in green bananas, cooked potatoes, and grains. These fiber types promote the growth of Firmicutes and Bacteroidetes. They also enhance SCFA production through bacterial fermentation. Polyphenols are widely present in fruits, whole grains, vegetables, and tea. Gut bacteria metabolize these compounds into bioactive metabolites. These metabolites exhibit anti-inflammatory and antioxidant properties. Polyphenols may also exert selective antimicrobial effects by suppressing pathogenic bacteria while supporting beneficial species [103].

Furthermore, emerging research suggests that periodic fasting and caloric restriction positively impact gut microbiota by promoting microbial diversity, reducing inflammation, and improving metabolic health through gut environment modulation.

### 5.3. Advanced Therapeutic Interventions

Fecal Microbiota Transplantation (FMT): FMT represents a comprehensive microbiota restoration approach involving the transfer of fecal material from healthy donors to recipients’ gastrointestinal tracts, introducing diverse microbial communities that outcompete pathogenic bacteria and restore beneficial microbes [104]. FMT has demonstrated remarkable clinical success with over 90% efficacy in treating recurrent *Clostridium difficile* infections and is being investigated for ulcerative colitis, Crohn’s disease, obesity, and type 2 diabetes. Early studies suggest that FMT may influence immunotherapy response in CRC patients by modulating the tumor microenvironment. However, CRC-specific interventional evidence remains limited, and the clinical role of FMT in this setting is still under investigation. Future microbiota-based interventions may need to move beyond single strains. In many cases, clinical effects may depend on bacterial communities that work together through cooperation or mutualism. This may be important for FMT, postbiotics, and engineered microbiome therapies [105]. A network-based view may also help explain why treatment responses differ across populations. In the future, functionally defined microbial consortia may prove more stable and reproducible than isolated strains.

Pharmacological agents: Pharmacological agents (e.g., rifaximin, metronidazole, clarithromycin, or amoxicillin) are increasingly being developed to selectively modulate gut microbiota for therapeutic purposes. Although broad-spectrum antibiotics can cause harmful dysbiosis, narrow-spectrum antibiotics target specific pathogens like *Helicobacter pylori* without disrupting overall microbiota balance. Novel microbiome-modulating drugs that enhance intestinal epithelial barrier function, regulate digestive enzymes, and modify bile acid metabolism are under development to promote healthier microbial profiles and treat obesity, diabetes, and colorectal cancer.

### 5.4. Personalized Medicine Approaches

Microbiota-based diagnostics and treatments: Microbiota-based diagnostics represent a rapidly expanding field leveraging gut microbiota’s impact on immune responses, metabolic processes, and neurological health. These diagnostics analyze patient microbiota composition using advanced techniques, including 16S rRNA sequencing for bacterial taxa identification, shotgun metagenomics for comprehensive microbial genome analysis, and metabolomics for profiling health-relevant microbial metabolites (SCFAs, secondary bile acids, tryptophan derivatives) [106]. This integrative approach enables personalized assessment of microbial health status and targeted therapeutic interventions. Microbiome-based diagnostics may be most useful when combined with current screening tools, rather than used alone. A practical future approach may be a stool-based model that combines selected bacterial markers with fecal hemoglobin or FIT-related thresholds. This may improve sensitivity and risk stratification. It may also be easier to integrate into existing screening programs [107,108]. However, its CRC-specific clinical utility will depend on further validation, reproducibility across cohorts, and compatibility with existing screening workflows.

Precision microbiome medicine: Recent advances in microbiome science are revolutionizing CRC prevention and treatment through personalized approaches. High-throughput sequencing technologies enable detailed characterization of individual microbial profiles, allowing clinicians to identify specific dysbiosis patterns associated with increased CRC risk. Some candidate bacteria should also be stated more clearly. *Fusobacterium nucleatum* is of particular interest because it has been linked to chemotherapy response and altered immunotherapy sensitivity [109,110]. By contrast, *Akkermansia muciniphila*, *Bifidobacterium* spp., and some SCFA-associated commensals are often discussed as potentially beneficial taxa in treatment response [111]. However, these organisms should still be regarded as candidate modulators rather than validated clinical biomarkers. Their effects may vary by tumor subtype, treatment type, and host background. At present, most evidence remains associative or early translational rather than interventional in CRC-specific clinical settings.

The emerging field of pharmacomicrobiomics has revealed critical interactions between gut microbiota and cancer therapeutics. Certain bacterial species enhance chemotherapeutic agent efficacy (like 5-FU conversion to active metabolites), whereas others mitigate drug-related toxicity. The gut microbiome plays a pivotal role in modulating immunotherapy responses, with specific microbes such as *Bifidobacterium* and *Akkermansia muciniphila* associated with improved outcomes to immune checkpoint inhibitors [112].

### 5.5. Regional and Cultural Considerations

Region-specific dietary recommendations based on local food cultures and microbiota profiles offer promising prevention strategies. Sichuan populations benefit from maintaining traditional fermented food consumption and reducing processed meat intake to optimize microbiota balance [113]. Mediterranean-style diets rich in fiber, polyphenols, and healthy fats consistently promote beneficial microbiota and reduce CRC risk across diverse populations [114]. Adapting these principles to local food preferences enhances intervention feasibility and sustainability, and dietary diversity through consuming varied whole, unprocessed foods supports microbial diversity and reduces metabolic syndrome risk through enhanced SCFA production [103].

These microbiota-targeted approaches highlight the potential of CRC prevention and treatment strategies tailored to microbial and dietary context. Current options include dietary modulation, probiotics, prebiotics, synbiotics, postbiotics, fecal microbiota transplantation, and microbiota-informed diagnostics. Each offers potential advantages, but each also has important limitations. Their clinical value is promising, yet CRC-specific interventional evidence remains limited, and broader application will require further validation, better standardization, and careful context-specific evaluation. At the same time, integration of microbiome profiling with clinical and genetic data may support more precise models for risk assessment, prevention, and treatment selection. However, these approaches are still developing and are not yet ready for routine clinical use in CRC. Effective CRC prevention is also unlikely to depend on a single universal dietary model. Region-adapted strategies are more likely to be practical and sustainable when they align with local food culture, microbial patterns, and long-term adherence. Although these strategies show considerable promise, CRC-specific interventional evidence remains limited, and many proposed applications should still be regarded as emerging rather than ready for routine clinical implementation.

## 6. Future Directions and Clinical Translation

Translating gut microbiota research into clinical practice for CRC prevention and treatment offers clear opportunities but also major challenges. The previous section summarizes current and emerging microbiota-targeted strategies. This section focuses instead on future directions, unresolved barriers, and the main requirements for clinical implementation. Progress has been made in biomarker discovery, risk stratification, microbiota-targeted intervention, and multi-omics integration. Even so, routine CRC-specific clinical use remains limited [115]. Important barriers include microbiome variability across populations, limited standardization, difficulty in establishing causality, safety concerns related to microbiota-based therapies, and the need for stronger prospective and multi-center validation studies. Several microbial signals remain of interest. *Parvimonas micra*, *Fusobacterium nucleatum*, and *Peptostreptococcus anaerobius* are strongly associated with CRC, whereas reduced levels of *Faecalibacterium prausnitzii* may indicate early disease or precancerous change [116]. Altered microbial metabolites—including secondary bile acids, indole derivatives, and SCFAs—also show promise as diagnostic indicators [117]. Metagenomic analysis of stool and blood samples may help detect dysbiosis-related genetic signatures [118], whereas liquid biopsies offer real-time monitoring through circulating microbial DNA [119].

However, many proposed biomarkers and interventions are still emerging and are not yet ready for routine clinical use. A major controversy in this field is the lack of reproducibility across studies. A bacterial signature reported in one cohort may not be confirmed in another. This may reflect differences in diet, antibiotic exposure, geography, tumor stage, host background, and study design. It is also often unclear whether a microbial signal is a causal driver, a passenger that expands after ecological disruption, or simply a context-dependent marker of the tumor environment. These uncertainties are further complicated by differences in sampling methods, sequencing platforms, bioinformatic pipelines, and analytical thresholds. Future progress will depend not only on identifying candidate microbes but also on improving reproducibility, clarifying causality, and developing standardized validation frameworks across diverse populations.

Coupled with artificial intelligence (AI)-driven data analysis, these tools may improve risk prediction and support more personalized diagnostic strategies by integrating microbiome data with clinical and genetic profiles. However, their performance, interpretability, and generalizability still require further evaluation [120]. However, most previous research in this field has centered on genomic biomarkers. Much of the work has also focused on AI-assisted instrumentation, colonoscopy, image analysis, and pathological assessment. In contrast, relatively few studies have applied artificial intelligence directly to metagenomic analysis. At present, there are relatively few studies on the application of AI to metagenomics in this field.

In parallel, synthetic biology is opening new therapeutic avenues by engineering microbes to deliver targeted treatments directly to tumor sites [121]. These “living therapeutics” are being explored as a way to deliver anti-inflammatory cytokines or anticancer agents within the tumor microenvironment. Their potential is promising, but their safety, stability, and CRC-specific clinical applicability remain to be established [122]. Genetically tailored microbial consortia are being developed as possible alternatives to fecal microbiota transplantation (FMT). They may offer more controlled interventions, but their safety, reproducibility, and clinical value in CRC remain uncertain. Additionally, postbiotics are gaining attention as potential standalone or adjunctive therapies. However, CRC-specific interventional evidence remains limited, and its clinical role has not yet been clearly defined [100].

A systems biology approach that integrates multi-omics data (e.g., microbiome, metabolome, and host transcriptome) is revealing complex interactions and identifying new therapeutic targets [123]. Research increasingly shows that microbiota-derived metabolites and microbial functional pathways can influence gene expression in CRC cells through epigenetic mechanisms, including DNA methylation- and chromatin-related regulation, thereby opening new possibilities for biomarker development and therapies targeting specific microbial pathways [63]. The gut–liver axis is another promising area, as understanding microbiota–hepatic interactions may lead to strategies for preventing CRC metastasis and improving treatment outcomes [124].

Despite progress, several barriers must be overcome for successful clinical translation. Microbiome variability across populations due to genetics, diet, and environment calls for precision medicine approaches rather than universal solutions [125]. Safety concerns around FMT and engineered probiotics require long-term evaluation to assess risks such as pathogen transmission and ecological disruption [126,127]. Establishing causal links between specific microbes and CRC remains difficult, highlighting the need for standardized protocols and longitudinal studies to identify optimal intervention windows.

To address these challenges, a growing number of clinical studies are exploring the role of the gut microbiota in CRC. These include both observational studies and early interventional trials. Researchers have tested dietary approaches (Table 6) and microbiota-targeted strategies such as prebiotics, probiotics, synbiotics, and postbiotics (Table 7). Some studies have also examined drugs that may modify the gut microbial environment (Table 8). Study outcomes vary widely. Common endpoints include microbiota modulation, postoperative complications, treatment tolerance, CRC recurrence, and the performance of screening or diagnostic tools. Methods such as RT-PCR, 16S rRNA sequencing, and metagenomic analysis are frequently used. Most registered studies have been carried out in Europe and Asia. Overall, these studies point to the potential clinical relevance of microbiota-centered approaches in CRC. Still, the evidence remains heterogeneous, and much of it is exploratory. Larger, better standardized, and multi-center studies are still needed before routine clinical use can be considered.

AI and machine learning are transforming data analysis, enabling more precise risk prediction and personalized treatment strategies [128]. Next-generation probiotics, optimized FMT protocols, and synthetic microbial consortia are at the forefront of therapeutic innovation. The integration of microbiota profiling with cancer immunotherapy is also showing promise, with microbial signatures being explored to enhance responses to immune checkpoint inhibitors. Key priorities include developing population-specific strategies to account for regional microbiome diversity, expanding research to include fungi and viruses in CRC pathogenesis, and conducting longitudinal studies to track microbiome changes over time.

Current evidence should also be interpreted with caution. Existing studies remain heterogeneous in terms of population background, diet, sample source, sequencing strategy, and analytical workflow [31,32,33,34,35,36,37,38,47,48,49,50,51,52,53,60,61,62,63,64,65]. Many reported microbial signatures are associative rather than causal, and CRC-specific intervention trials are still limited. In addition, the roles of the gut mycobiome and virome remain underexplored, and most published studies are cross-sectional rather than longitudinal. These issues continue to restrict reproducibility, mechanistic interpretation, and clinical translation [67,76,77,78,79,80,81,82,83,84,85,92,93,94,95,96,97,98,99,100,101,102,103,104,105,106,107,108,109].

The Sichuan regional study offers important insights for CRC prevention strategies, demonstrating how traditional dietary patterns can shape microbial ecosystems that may mitigate cancer risk through multiple mechanisms. These include enhanced SCFA production, maintenance of mucosal barrier integrity, and immunomodulation. However, the coexisting high intake of processed meats presents a counterbalancing cancer risk, underscoring the importance of considering overall dietary patterns rather than focusing on single food components when examining microbiota–CRC relationships. This regional perspective highlights the complex interplay between diet, gut microbiota, and colorectal health, providing valuable information for developing targeted dietary interventions for CRC prevention.

Regulatory frameworks must ensure the safety and efficacy of novel microbial therapies, while cost-effective implementation and provider education are essential for broad adoption. Preserving traditional diets amid urbanization and fostering international collaboration will also be critical to ensuring global equity in accessing microbiota-based innovations. The convergence of microbiome science with precision oncology represents a major shift in CRC prevention and treatment. While challenges remain in standardization, safety, and implementation, the potential to reduce CRC incidence through targeted microbial interventions is substantial. By combining traditional knowledge with cutting-edge science and fostering global cooperation, we can harness the therapeutic power of the microbiome to transform colorectal cancer care.

## 7. Conclusions

In conclusion, the gut microbiota has emerged as a key contributor to CRC pathogenesis through its effects on host immunity, inflammatory signaling, and metabolic regulation. Regional dietary patterns further shape these microbial ecosystems and may influence CRC risk. In Sichuan, traditional fermented foods and plant-rich dietary practices may support beneficial microbiota with protective potential. However, rapid urbanization and dietary westernization are increasingly disrupting these microbial communities.

Mechanistic studies have shown that pathogenic bacteria may promote tumorigenesis by activating pro-inflammatory and oncogenic pathways, whereas beneficial commensals may counteract these effects by preserving barrier integrity and producing SCFAs. These findings provide a strong rationale for the development of microbiota-based strategies in CRC management, including precision probiotics, microbiota-guided immunotherapy, and AI-assisted risk prediction models.

Despite these advances, important challenges remain, particularly in the standardization, safety evaluation, and population-specific adaptation of microbiota-based diagnostics and therapies. Nevertheless, the integration of microbiome science with precision oncology offers a promising framework for future CRC prevention and treatment. By bridging traditional dietary knowledge with modern biomedical innovation, the gut microbiota may be developed not only as a biomarker but also as a modifiable therapeutic target for reducing the global burden of colorectal cancer.

## Figures and Tables

**Table 1:** Microbial drivers and guardians in colorectal carcinogenesis. Key bacterial species involved in CRC development, distinguishing between pathogenic species that promote carcinogenesis and protective species that inhibit tumor formation. The mechanisms of action illustrate how specific bacteria influence molecular pathways, inflammation, and cellular processes.

Bacterial Species	Phylum	Role in CRC	Mechanisms of Action	Clinical/Therapeutic Implication	Ref.
Pathogenic Species					
*Fusobacterium nucleatum*	*Fusobacteria*	Pro-carcinogenic	FadA adhesin binding to E-cadherin NF-κB activation Immune evasion	Personalized microbiome-based therapies Targeted molecular interventions (against DHX15 or the *F. nucleatum*-DHX15 interaction)	[31]
*Bacteroides fragilis*	*Bacteroidetes*	Pro-carcinogenic	Bft toxin production DNA damage induction Wnt/β-catenin activation Inflammation promotion	Microbial biomarker for diagnosis	[32]
*Enterococcus faecalis*	*Firmicutes*	Pro-carcinogenic	ROS production DNA damage Chronic inflammation	Potential diagnosis biomarker or therapeutic target	[33]
*Escherichia coli* (pks+)	*Proteobacteria*	Pro-carcinogenic	Colibactin toxin production Genotoxic effects Biofilm formation	Potential biomarker for CRC risk assessment Targeting pks+ *E. coli* or inhibiting colibactin biosynthesis	[34]
Protective Species					
*Faecalibacterium prausnitzii*	*Firmicutes*	Anti-carcinogenic	Butyrate production Anti-inflammatory effects Barrier integrity maintenance	Next-generation probiotic candidate for CRC prevention and gut health maintenance	[35]
*Lactobacillus* spp.	*Firmicutes*	Anti-carcinogenic	Lactic acid production Pathogen inhibition Immune modulation	Adjunctive therapy or preventive agents in CRC management	[36]
*Bifidobacterium* spp.	*Actinobacteria*	Anti-carcinogenic	SCFAs production Immune regulation Vitamin synthesis	Probiotic adjuvant for CRC prevention and treatment	[37]
*Akkermansia muciniphila*	*Verrucomicrobia*	Anti-carcinogenic	Mucin degradation Barrier strengthening Metabolic regulation	Epigenetic suppression of colorectal cancer Enhancement of anti-tumor immunity Safe and targeted delivery through nanoparticle formulation	[38]

Abbreviation: ROS, reactive oxygen species; DNA, deoxyribonucleic Acid; SCFAs, short-chain fatty acids; NF-κB, Nuclear Factor kappa-light-chain-enhancer of activated B cells; pks+, polyketide synthase-positive.

**Table 2:** Microbial metabolites in colorectal cancer. Major categories of microbial metabolites and their differential effects on colorectal health. Beneficial metabolites, particularly short-chain fatty acids, provide protective effects against CRC through anti-inflammatory and anti-proliferative mechanisms. Harmful metabolites from dysbiotic bacteria promote carcinogenesis through DNA damage, inflammation, and barrier dysfunction. Additional relevant studies are summarized in Supplementary Table S2.

Metabolite Category	Specific Compounds	Producing Bacteria	Effects on Colon	Impact on CRC Risk	Clinical/Therapeutic Implication	Ref.
Beneficial Metabolites						
Short-chain fatty acids	Butyrate	*F. prausnitzii*, *Clostridium* spp.	Primary energy source for colonocytes HDAC inhibition Anti-inflammatory Apoptosis induction	↓ Risk (protective)	With chemopreventive potential Targeted delivery Combination therapies Microbiome modulation	[53]
	Propionate	*Bacteroides*, *Prevotella*	Gluconeogenesis regulation Immune modulation	↓ Risk (protective)	Therapeutic metabolite or adjuvant in CRC prevention and treatment	[51]
	Acetate	Various Firmicutes	Lipid synthesis pH reduction	↓ Risk (protective)	Improving gut homeostasis and reducing inflammation Potential adjunctive therapeutic value	[54]
Vitamins	Vitamin K2	*E. coli*, *Bacteroides*	Coagulation support Bone metabolism	Neutral/protective	Enhance the efficacy of conventional chemotherapeutics Potential adjunctive therapeutic agent	[55]
	B vitamins	*Lactobacillus*, *Bifidobacterium*	Metabolic cofactors DNA synthesis	Neutral/protective	Protective nutrients Supportive agents	[56]
Harmful Metabolites						
Secondary bile acids	Deoxycholic acid	*Clostridium* spp., *Eubacterium*	DNA damage Oxidative stress Inflammation	↑ Risk (carcinogenic)	Biomarker for high-risk individuals Dietary interventions Targeted therapies	[57]
	Lithocholic acid	Various anaerobes	Cytotoxicity Barrier dysfunction	↑ Risk (carcinogenic)	Biomarker potential Dietary modifications Bile acid sequestrants	[58]
Protein fermentation products	Ammonia	*Clostridium*, *Bacteroides*	Epithelial toxicity Barrier disruption	↑ Risk (toxic)	Biomarker potential Dietary interventions (Reduced animal protein) Microbiome modulation	[26]
	Hydrogen sulfide	*Desulfovibrio* spp.	Genotoxicity Inflammation	↑ Risk (genotoxic)	Biomarker potential Dietary interventions Combination therapies	[59]
	p-Cresol	*Clostridium difficile*	Oxidative stress DNA damage	↑ Risk (carcinogenic)	Risk Stratification Poor Prognosis Indicator Dietary interventions Microbiome modulation	[60]
Polyamines	Putrescine, Spermidine	Various bacteria	Cell proliferation Tumor growth (excess)	↑ Risk (high levels)	Diagnosis biomarker Therapeutic target Microbiome modulation Engineered combination therapies	[61,62]

Abbreviations: HDAC = histone deacetylase. ↑ = increased; ↓ = decreased.

**Table 3:** Regional microbiome signatures and dietary influences in CRC susceptibility. The data illustrates how traditional diets rich in fiber and fermented foods correlate with beneficial microbial profiles.

Region/Population	Dominant Microbial Taxa	Key Dietary Components	Protective/Risk Factors	Ref.
Western (US/Europe)	*Bacteroides* spp., Proteobacteria	High fat, processed foods, low fiber	^a^ ↓ Microbial diversity, ↓ SCFA production	[67]
Sichuan (China)	*Lactobacillus* spp., *Bifidobacterium* spp., and *SCFA*-associated commensals (representative/hypothesized)	Fermented foods, spices, vegetables	^b^ ↑ SCFA production, fermented foods	[68]
Japan	*Bifidobacterium*, *Prevotella*, *B. plebeius*	Fermented soy, seaweed, fish	Seaweed polysaccharides, miso/natto	[69]
Rural Africa	*Prevotella* spp., Firmicutes	High fiber, plant-based	↑ Microbial diversity, fiber fermentation	[70]
Mediterranean	*Prevotella*, *F. prausnitzii*	Olive oil, vegetables, fish	Polyphenols, healthy fats	[84]
Urban China	*Transitioning profile*	Westernizing diet	Dietary transition effects	[75]

Abbreviations: SCFA, short-chain fatty acids. ^a^ ↑, increased; ^b^ ↓, decreased.

**Table 4:** From gut dysbiosis to clinical translation in colorectal cancer: representative microbial patterns, mechanisms, biomarkers, interventions, and evidence. References: Enrichment of pro-carcinogenic taxa [31,32,33,34,39,40,41,42,43,44,45,46]; depletion of beneficial SCFA-producing taxa [35,36,37,38,47,48,49,50,51,52,53]; carcinogenic metabolite-associated imbalance [26,48,50,51,52,53,54,55,56,57,58,59]; biofilm-associated mucosal dysbiosis [31,34,39,40]; microbiota-associated variability in treatment response [38]; region- and diet-associated dysbiosis patterns [60,61,62,63,64,65].

Dysbiosis Pattern	Key Microbes	Main Mechanisms	Candidate Biomarkers	Intervention Opportunities	Evidence Level
Enrichment of pro-carcinogenic taxa	*Fusobacterium nucleatum*, enterotoxigenic *Bacteroides fragilis*, pks+ *Escherichia coli*, *Enterococcus faecalis*	Chronic inflammation, epithelial injury, immune evasion, genotoxicity, activation of NF-κB, IL-6/STAT3, and Wnt/β-catenin signaling	Pathogenic taxa, toxin genes, inflammatory mediators, tissue-associated microbial signatures	Dietary regulation, targeted microbiota modulation, probiotic or synbiotic support	Strong preclinical; moderate observational clinical evidence
Depletion of beneficial SCFA-producing taxa	*Faecalibacterium prausnitzii*, *Lactobacillus* spp., *Bifidobacterium* spp., *Akkermansia muciniphila*	Reduced SCFA production, impaired barrier integrity, weakened anti-inflammatory signaling, altered immune homeostasis	Reduced SCFA levels, loss of butyrate producers, reduced microbial diversity	High-fiber diet, prebiotics, probiotics, synbiotics, postbiotics, microbiota restoration	Moderate observational; limited interventional CRC-specific evidence
Carcinogenic metabolite-associated imbalance	Secondary bile acids, hydrogen sulfide, ammonia, p-cresol, excess polyamines	Oxidative stress, DNA damage, epithelial toxicity, barrier dysfunction, pro-carcinogenic metabolic reprogramming	Metabolomic profiles, bile acid signatures, SCFA depletion, sulfur metabolite levels	Dietary modification, metabolite-guided risk stratification, microbiota-targeted nutritional strategies	Moderate mechanistic; emerging translational evidence
Biofilm-associated mucosal dysbiosis	Polymicrobial biofilms including *F. nucleatum*, *Bacteroides* spp., and pks+ *E. coli*	Mucosal invasion, persistent inflammatory signaling, barrier disruption, tumor-promoting microenvironment	Biofilm detection, tissue-associated microbial signatures, colonoscopy-linked microbial profiling	Early detection, anti-biofilm strategies, mucosal microbiota monitoring	Strong mechanistic; limited clinical implementation
Microbiota-associated variability in treatment response	*F. nucleatum*, SCFA-producing commensals, Akkermansia muciniphila and other immunomodulatory taxa	Altered chemotherapy response, immune modulation, inflammatory signaling, and drug metabolism	Stool metagenomics, response-associated microbial signatures, treatment-related microbial shifts	Personalized microbiome profiling, adjunct microbiota modulation, pharmacomicrobiomics-guided strategies	Emerging clinical evidence
Region- and diet-associated dysbiosis patterns	Variation in *Bacteroides*, *Prevotella*, *Lactobacillus*, *Bifidobacterium*, and SCFA-producing taxa	Diet–microbiota–metabolite interactions influencing CRC susceptibility	Region-specific microbial profiles, SCFA patterns, bile acid signatures	Precision nutrition, microbiota-informed prevention, region-adapted dietary guidance	Mostly observational and inferential evidence

Abbreviations: CRC, colorectal cancer; SCFA, short-chain fatty acid; NF-κB, nuclear factor kappa B; IL-6, interleukin-6.

**Table 5:** Major microbiota-targeted preventive and therapeutic strategies in CRC: representative approaches, mechanisms of action, advantages, and limitations.

Intervention Type	Specific Approaches	Mechanism of Action	Advantages	Limitations
Microbial Supplementation (Probiotics) [89]	Single-strain: *Lactobacillus rhamnosus* *Bifidobacterium longum* *Bifidobacterium breve* *Saccharomyces boulardii*	Restoration of microbiota balance SCFA production Immune modulation Pathogen exclusion Gut barrier enhancement	Well-established safety Easy administration Cost-effective	Variable efficacy Strain-specific effects Limited colonization
Microbial Supplementation (Prebiotics) [90]	Fructooligosaccharides (FOS) Inulin Galactooligosaccharides (GOS) Resistant starch Dietary fibers	Selective stimulation of beneficial bacteria SCFAs enhancement Microbiota diversification	Natural compounds Broad microbiota impact Easy dietary integration	Individual variation GI side effects Slow onset of action
Microbial Supplementation (Synbiotics) [91]	Probiotic + prebiotic combinations	Enhanced probiotic survival Synergistic microbiota modulation More durable effects	Synergistic benefits Improved formulation stability Potential enhanced efficacy	Higher costs Formulation complexity Efficacy varies by combination
Dietary Pattern Interventions (Fiber and polyphenol-rich diets) [92]	High-fiber intake Polyphenol-rich foods Mediterranean diet Fermented food consumption Reduced processed meat	Growth of beneficial microbes SCFA production increase Anti-inflammatory effects Suppression of pathogens	Natural, sustainable Culturally adaptable Broad health benefits	Requires long-term adherence Individual dietary variability Potential compliance challenges
Dietary Pattern Interventions (Fasting and caloric restriction) [93,94]	Intermittent fasting Time-restricted eating Caloric restriction	Increases microbiota diversity Lowers inflammation Improves metabolic homeostasis Modulates gut environment	Natural and low-cost Multiple systemic benefits	Tolerability varies Compliance issues Long-term safety/data still emerging
Faecal Microbiota Transplantation [95]	Fresh or frozen donor stool Standardized screening Capsule or colonoscopy delivery	Reconstitution of microbial ecosystem Rapid recolonization Restoration of immune and barrier functions Enhances immunotherapy outcomes in CRC	Effective for CDI Fast-acting Broad ecosystem reset	Risk of pathogen transmission Safety/regulatory concerns Need for donor matching Limited CRC-specific data
Pharmacological agents [96]	Narrow-spectrum antibiotics Bile acid modulators Microbiome-directed drugs Gut barrier enhancers	Pathogen suppression Modulation of microbial composition Barrier restoration	Precision control Well-defined mechanisms	Resistance development Off-target effects Drug-specific toxicity High development cost
Microbiota-based diagnostics and treatments [97]	16S rRNA gene sequencing Shotgun metagenomics Metabolomics AI/ML modelling	Early CRC detection Risk prediction Therapy monitoring Microbial signature identification	Non-invasive Personalized High potential for early intervention	Standardization challenges Technical interpretation Cost and infrastructure barriers
Personalized microbiome medicine [98]	Individual microbiota profiling Precision therapeutics Pharmacomicrobiomics AI-guided interventions	Tailored interventions Optimized drug response Reduced side effects Enhanced efficacy	Personalized outcomes Precision targeting Lower risk of adverse effects	Costly Technically complex Limited large-scale clinical validation
Regional and cultural interventions [99]	Traditional fermented foods Mediterranean-style adaptations Promotion of local diets Reduced ultra-processed foods	Supports region-specific microbiota Enhances traditional beneficial strains Integrates with sustainable food culture	Culturally relevant Community-based Affordable	Urbanization pressures Implementation variability Educational outreach needed

Abbreviations: CRC, colorectal cancer; SCFA, short-chain fatty acid; CDI, *Clostridioides difficile* infection; AI, artificial intelligence; ML, machine learning; FOS, fructooligosaccharides; GOS, galactooligosaccharides.

**Table 6:** Dietary interventions in clinical trials targeting the gut microbiome in CRC. This table summarizes selected representative clinical trials evaluating dietary interventions targeting the gut microbiome in CRC. Because the number of registered studies is increasing rapidly and study designs remain heterogeneous, only representative trials are included in the main text. Additional relevant studies are summarized in Supplementary Table S1.

Study Title	Brief Summary	Interventions	Study Type	Study Status	NCT Number
Microbiome Testing for the Screening of CRC	Gut microbiome alterations offer promise as non-invasive biomarkers. This study aims to develop a microbiome-based diagnostic tool for detecting CRC and advanced adenomas in FIT-positive individuals aged 50–74.	No intervention	Observational	Recruiting	NCT06588166
Microbiome-based Diagnostic Tool for the Screening of CRC (GUILTI)	to develop a microbiome-based tool to detect CRC and advanced adenomas in FIT-positive individuals aged 50–69.	No intervention	Observational	Recruiting	NCT06738173
Gut Microbiome in CRC	This is a pilot feasibility study designed to investigate the alterations in the gut microbiome that occur during the course of treatment for CRC	No intervention	Observational	Completed	NCT04054908
Characteristics of Gut Microbiota in Patients With Colon Cancer of Different TCM Syndromes	This study tracks CRC patients before and after surgery, linking shifts in TCM syndromes and constitutions (via questionnaires) with gut-microbiota changes (via next-generation sequencing).	No intervention	Observational	Completed	NCT03892252
Intestinal Flora Differences Between CRC Patients and Healthy Individuals	This case–control study compared gut microbiota between 36 CRC patients and 25 healthy controls. CRC patients showed reduced beneficial bacteria (e.g., Lactobacillus) and increased harmful/neutral taxa (e.g., Staphylococcus), with dysbiosis worsening from stage I to III.	No intervention	Observational	Completed	NCT06875648
Study of Gut Microbiome and Colorectal Tumors	Gut microbiota were assessed in 540 colonoscopy-screened adults by 16S rRNA gene sequencing of stool samples. Investigators compared gut microbiota diversity, overall composition, and normalized taxon abundance	No intervention	Observational	Completed	NCT03297996
Gut Microbiome and CRC	In Egypt, CRC ranks seventh overall, third in males, and fifth in females. Microbiome dysbiosis may contribute to CRC pathogenesis and offer a potential therapeutic target.	No intervention	Observational	Completed	NCT06748339
CRC Associated Host and Microbiome Study	Recruit healthy, precancer and CRC patients and record necessary information of demographic and other messages. All the volunteers were asked to provide samples including stool, blood, urine and tissues.	No intervention	Observational	Recruiting	NCT03998644
Effects of Red Ginseng on Gastrointestinal Symptoms and Microbiota After Surgery for Gastrointestinal Cancer	Gastrointestinal cancer surgery often leads to symptoms like weight loss and digestive issues, likely linked to gut microbiota changes. This study evaluates whether red ginseng, with its prebiotic effects, can improve gut microbiota, gastrointestinal symptoms, and nutritional status after surgery.	Diet (Red Ginseng)	Interventional	Completed	NCT06561516
Calcium: Magnesium Balance, Microbiota, and Necroptosis and Inflammation	To explore the gut microbiota’s role in this association, a double-blind 2 × 2 factorial RCT (NCT01105169) will assess whether optimizing the Ca: Mg ratio to 2.3 alters microbial abundance related to TRPM7 genotype and metachronous polyp risk across stool, swab, and tissue samples.	Dietary supplement: Magnesium glycinate and Placebo	Interventional	Completed	NCT04229992
Ginger and Gut Microbiome (GINGER)	Estimate the impact of a 6-week daily intake of 2000 mg of ginger extract on the composition of the gut microbiome using a randomized placebo-controlled double-blinded design, i.e., examine the change in microbiome over time within and between the subjects.	Dietary supplement: Ginger extract and Placebo	Interventional	Completed	NCT03268655
Dietary Supplement on the Intestinal Microbiota in Patients with Colon Cancer	Effect of a dietary supplement with antioxidant and anti-inflammatory properties on the intestinal microbiota in patients with colon cancer. Randomized placebo-controlled clinical trial. Ter atrophic study	DCOOP Product, Hydroxytyrosol extract and Indukern product, Curcumin and selenium extract	Interventional	Completed	NCT05472753
Study to Assess Colonic Microbiota Changes in Response to Energy Drink Consumption	This study will investigate whether short-term daily energy drink consumption results in an increase in hydrogen sulfide-producing bacteria in adults 18–40 years old.	Dietary supplement: Energy drink	Interventional	Completed	NCT06137248
Microbiome Test for the Detection of Colorectal Polyps and Cancer	This study aims to evaluate the sensitivity, specificity, and accuracy of the Metabolomics colon polyp and CRC assay for the non-invasive detection of colorectal polyps and cancer.	No intervention	Observational	Completed	NCT02141945
Obesity, Iron Regulation and CRC Risk	This study will conduct a crossover feeding trial comparing three diets—high-iron typical American, low-iron typical American, and high-iron Mediterranean—to assess their effects on gut microbiota and inflammation.	High heme iron diet, Low iron diet and Plant-based high non-heme iron diet	Interventional	Completed	NCT03548948
*Fusobacterium Nucleatum* at CRC Sites	This study investigates whether the oral cavity serves as a reservoir for *Fusobacterium nucleatum* in CRC patients. It will assess the relationship between oral, gut, and tumor colonization by *F. nucleatum*, along with dietary and microbiome factors.	Other: biopsy	Interventional	Recruiting	NCT05945082
The Impact of Palm Date Intake on Colon Health Biomarkers	This study investigates the prebiotic effects of date fruit in healthy individuals using a 21-day crossover design with a 14-day washout. Fecal and blood samples will be analyzed to evaluate metabolic responses, microbiota shifts, and chronic disease biomarkers.	Date fruit—Ajwa variety and Maltodextrin/Dextrose	Interventional	Completed	NCT02288611
Investigation of the Role of the Microbiome in the Pathogenesis of Colorectal Adenoma and Carcinoma	This study aims to elucidate host–microbiome interactions that drive adenoma formation and CRC progression. Saliva, stool, and colon biopsy samples will be collected from patients alongside dietary, lifestyle, and medical history data. Host and microbial genomes and transcriptomes will be analyzed in parallel.	No intervention	Observational	Completed	NCT02947607

Abbreviations: TCM, traditional Chinese medicine; NCT, national clinical trial; RCT, randomized controlled trial.

**Table 7:** Clinical trials of microbiota-directed interventions (prebiotics, probiotics, synbiotics, and postbiotics) in CRC. This table summarizes selected representative clinical trials evaluating microbiota-directed interventions, including prebiotics, probiotics, synbiotics, and postbiotics, in CRC. Because the number of registered studies is increasing rapidly and study designs remain heterogeneous, only representative trials are included in the main text. Additional relevant studies are summarized in Supplementary Table S2.

Study Title	Brief Summary	Interventions	Study Type	Study Status	NCT Number
Prebiotic-based intervention					
Prebiotic Effect of Eicosapentaenoic Acid Treatment for CRC Liver Metastases	This study, linked to the EMT2 trial (NCT03428477), investigates how the omega-3 fatty acid EPA may prevent cancer recurrence after liver surgery for colorectal metastases. By analyzing stool, urine, blood, and tumor samples, it aims to uncover microbiome and immune mechanisms behind EPA’s effects and identify patients most likely to benefit, supporting personalized treatment approaches.	Drug: Icosapent Ethyl Oral Capsule	Interventional	Completed	NCT04682665
Probiotic-based intervention					
Impact of Probiotics on the Intestinal Microbiota	This study aims to evaluate the effects of probiotic administration (*Saccharomyces boulardii*) in patients undergoing colorectal resection compared to standard care. Outcomes include: (1) modulation of intestinal microbiota and (2) postoperative outcomes.	Dietary supplement: *Saccharomyces boulardii*	Interventional	Completed	NCT01609660
Impact of Probiotics in Modulation of Intestinal Microbiota	The investigators would study about impact of the administration of probiotics in the intestinal mucosa of patients undergoing resection colic, by evaluating cytokine profile by quantitative real-time PCR.	Dietary supplement: *Saccharomyces boulardii*	Interventional	Completed	NCT01895530
Microbiota Implementation to Reduce Anastomotic Colorectal Leaks	Aim of this study is to implement the intestinal microbiota by perioperative administration of probiotics, oral antibiotics and low-volume mechanical preparation.	Probiotics, oral antibiotics and mechanical preparation	Interventional	Completed	NCT05164887
Using Probiotics to Reactivate Tumor Suppressor Genes in Colon Cancer	This study aims to determine whether probiotic supplementation can positively influence colon cancer-associated microbiota and epigenetic changes. Participants will receive two daily ProBion Clinica tablets containing *Bifidobacterium lactis* Bl-04, *Lactobacillus acidophilus* NCFM, and inulin.	Dietary supplement: ProBion Clinica	Interventional	Completed	NCT03072641
Probiotics In CRC Patients	This double-blind, randomized trial assesses bacterial colonization at surgery (Day 0), impacts on gut microbiota and immune response, and explores dose-dependent colonization of *Lactobacillus acidophilus La1* and its influence on microbial and immunological outcomes.	Procedure: Probiotics (La1, BB536) and placebo	Interventional	Completed	NCT00936572
An Evaluation of Probiotic in the Clinical Course of Patients With CRC	This study investigates whether probiotic functional foods can reduce inflammation and improve symptoms in CRC patients by modulating the gut microbiome.	Dietary supplement: Probiotic	Interventional	Completed	NCT03782428
Synbiotic-based intervention					
Effect of Synbiotic Supplementation on the Prevention of Mucositis in Cancer Patients Undergoing Chemotherapy	This randomized clinical trial evaluates whether pre-chemotherapy synbiotic supplementation can reduce mucositis and diarrhea in CRC patients treated with capecitabine, aiming to improve gut health and quality of life.	Dietary supplement: Symbiotic	Interventional	Recruiting	NCT06576986
Action of Synbiotic on Irradiated GI Mucosa in Rectal Cancer Treatment	The aim of this study is to investigate how bacteria and fiber interact with the epithelial cells of the gastrointestinal mucosa to reduce inflammation and to diminish tissue damage caused by radiation therapy.	Oat bran and blueberry husks	Interventional	Completed	NCT03420443
Postbiotic-based intervention					
Postbiotics for Mitigation of Postoperative Dysbiosis in Colon Cancer Surgery	This study assesses the efficacy of postbiotic supplements in reducing gut dysbiosis after colon cancer surgery by measuring changes in fecal Shannon Diversity Index (SDI) from baseline to postoperative timepoints at 2 weeks post-surgery.	Dietary supplement: PoZibio and Inert placebo	Interventional	Not Yet Recruiting	NCT07050485

**Table 8:** Drug-mediated modulation of the gut microbiome in CRC clinical trials. Additional relevant studies are summarized in Supplementary Table S3.

Study Title	Brief Summary	Interventions	Study Type	Study Status	NCT Number
Coffee and Metabolites Modulating the Gut Microbiome in CRC	This study is assessing the effects of 6-g daily use of freeze-dried instant coffee on liver fat and fibrosis and the gut microbiome and metabolome in patients who have completed routine treatment (including surgery, chemotherapy and radiotherapy) for stage I–III CRC.	Drug: Nestlé NESCAFÉ^®^ TASTER’S CHOICE^®^ House Blend capsule and Placebo	Interventional	Recruiting	NCT05692024
Omega 3 Fatty Acids in CRC Prevention in Patients With Lynch Syndrome	This is a pilot study aimed at assessing the effects of moderate dose omega-3-acid ethyl esters capsules (generic Lovaza) on molecular and intestinal microbiota changes in participants at high risk for CRC. The study will be a single-arm, open-label study.	Drug: Omega-3 fatty acid ethyl esters (2 g)	Interventional	Unknown	NCT03831698
OMega-3 Fatty Acid for the Immune Modulation of CRC	This trial evaluates whether daily 4 g marine omega-3 (VASCEPA) alters the tumor immune environment and gut microbiome in CRC patients before surgery, using a double-blind, placebo-controlled design.	Drug: AMR101 (VASCEPA, icosapent ethyl)	Interventional	Withdrawn	NCT03661047
Study of Berberine Hydrochloride in Prevention of Colorectal Adenomas Recurrence	In recent years, anticancer activity of berberine hydrochloride has been explored. The aim of this study is to investigate the effect of berberine hydrochloride on the recurrence of colorectal adenomas.	Drug: Berberine hydrochloride	Interventional	Completed	NCT02226185

## Data Availability

No new data were created or analyzed in this study. Data sharing is not applicable to this article.

## Supplementary Materials

The following supporting information can be downloaded at: https://www.mdpi.com/article/10.3390/cancers18111693/s1, Table S1. Dietary intervention in clinical trials targeting gut microbiome in CRC; Table S2. Microbiota-directed (prebiotic, probiotic, synbiotic, postbiotic) intervention for CRC in clinical trial; Table S3. Drug-mediated modulation of Gut-microbiome of CRC in clinical trial.

## Abbreviations

The following abbreviations are used in this manuscript:

AI	artificial intelligence
CRC	colorectal cancer
EPA	eicosapentaenoic acid
FIT	fecal immunochemical test
FMT	fecal microbiota transplantation
FOS	fructooligosaccharides
GOS	galactooligosaccharides
HFD	high-fat diet
HLA	human leukocyte antigen
IBD	inflammatory bowel disease
IBS	irritable bowel syndrome
IL-1β	interleukin-1 beta
IL-6	interleukin-6
MAC	microbiota-accessible carbohydrates
ML	machine learning
NF-κB	nuclear factor kappa B
PM2.5	particulate matter with an aerodynamic diameter ≤ 2.5 μm
RCT	randomized controlled trial
ROS	reactive oxygen species
RT-PCR	reverse transcription polymerase chain reaction
SCFA	short-chain fatty acid
SCFAs	short-chain fatty acids
TCM	traditional Chinese medicine
TLR	toll-like receptor
TMAO	trimethylamine N-oxide
TNF-α	tumor necrosis factor alpha
5-FU	5-fluorouracil
