# Supplementary material for: Gut Microbiota in Colorectal Cancer: Mechanistic Insights, Clinical Strategies, and a Regional Perspective with a Focus on Sichuan, China
Source: Cancers (Basel). 2026 May 22;18(11):1693. doi: 10.3390/cancers18111693 (PMC13255713; doi:10.3390/cancers18111693)
Supplement: Supplementary file 1 [file cancers-18-01693-s001.zip › cancers-4248829-supplementary.pdf]

**Table S1.** Dietary intervention in clinical trials targeting gut microbiome in CRC.

| Study Title | Brief Summary | Interventions | Study Type | Study Status | NCT Number |
|---|---|---|---|---|---|
| Gut Microbiota and CRC | The study aims to develop a screening test for individuals at risk of CRC (aged over 50), with improved sensitivity and prognostic accuracy compared to the current standard method, faecal occult blood testing. | No intervention | Observational | Unknown | NCT04662853 |
| The Impact of Immunonutrition on Gut Microbiota-related Aspects in CRC and Gastric Cancer Patients | Enteral immunomodulating nutrition modulates the gut microbiota and enhances intestinal barrier integrity in gastric and colorectal cancer patients during the perioperative period, reducing postoperative complications and preoperative treatment-related diarrhoea. | Impact Oral Nestl Health Science, Cubitan Nutricia, Nutridrink Nutricia, Resource 2.0 Nestl Health Science | Interventional | Unknown | NCT04980950 |
| Gut Microbiota Prediction of Metachronous CRC | CRC patients are at high risk for metachronous adenomas. This study aims to develop a microbiota-based model to predict postoperative CRC | No intervention | Observational | Unknown | NCT03383159 |

| | | | | | |
|---|---|---|---|---|---|
| Microbiome Testing for the Screening of CRC | Gut microbiome alterations offer promise as non-invasive biomarkers. This study aims to develop a microbiome-based diagnostic tool for detecting CRC and advanced adenomas in FIT-positive individuals aged 50–74. | No intervention | Observational | Recruiting | NCT06588166 |
| Microbiome-based Diagnostic Tool for the Screening of CRC (GUILTI) | to develop a microbiome-based tool to detect CRC and advanced adenomas in FIT-positive individuals aged 50–69. | No intervention | Observational | Recruiting | NCT06738173 |
| Gut Mucosal Microbiota is Associated with CRC Relapse | This study hypothesizes that gut microbiota may influence CRC relapse after curative treatment. It aims to identify microbiota differences between relapsed and non-relapsed patients and develop microbiota-based, patient-centered surveillance protocols. | No intervention | Observational | Unknown | NCT03385213 |
| Analysis of Intestinal Microflora Combined with DNA Methylation in Stool to Detect CRC | This study aims to establish a non-invasive, microbiome-based screening model by analyzing 16S rRNA and DNA methylation in faecal samples from CRC patients and healthy controls, with validation through multicenter studies. | No intervention | Observational | Unknown | NCT04302363 |
| Gut Microbiome in CRC | This is a pilot feasibility study designed to investigate the alterations in the gut microbiome that occur during the course of treatment for CRC | No intervention | Observational | Completed | NCT04054908 |

| | | | | | |
|---|---|---|---|---|---|
| Characteristics of Gut Microbiota in Patients With Colon Cancer of Different TCM Syndromes | This study tracks CRC patients before and after surgery, linking shifts in TCM syndromes and constitutions (via questionnaires) with gut-microbiota changes (via next-generation sequencing). | No intervention | Observational | Completed | NCT03892252 |
| Modified MAC Diet and Gut Microbiota in CRC Patients | This study evaluates gut microbiota changes over three weeks of a modified MAC diet versus a conventional diet in post-surgical early-stage CRC patients. | Modified MAC diet (3-weeks) | Interventional | Unknown | NCT05039060 |
| Intestinal Flora Differences Between CRC Patients and Healthy Individuals | This case-control study compared gut microbiota between 36 CRC patients and 25 healthy controls. CRC patients showed reduced beneficial bacteria (e.g., Lactobacillus) and increased harmful/neutral taxa (e.g., Staphylococcus), with dysbiosis worsening from stage I to III. | No intervention | Observational | Completed | NCT06875648 |
| A Cross-sectional Study on the Association of Peptidoglycan Fragments Derived from Gut Microbiota With CRC | This Observational study investigates whether peptidoglycan fragments from gut microbiota are associated with CRC progression. Participants will provide blood, feces, and colon tissue samples for analysis. | No intervention | Observational | Recruiting | NCT06379412 |
| Study of Gut Microbiome and Colorectal Tumours | Gut microbiota were assessed in 540 colonoscopy-screened adults by 16S rRNA gene sequencing of stool samples. Investigators compared gut microbiota diversity, overall composition, and normalized taxon abundance | No intervention | Observational | Completed | NCT03297996 |

| | | | | | |
|---|---|---|---|---|---|
| Gut Microbiome and CRC | In Egypt, CRC ranks seventh overall, third in males, and fifth in females. Microbiome dysbiosis may contribute to CRC pathogenesis and offer a potential therapeutic target. | No intervention | Observational | Completed | NCT06748339 |
| Serial Gut Microbiome and Bacterial Gene Markers Changes After Endoscopic Resection of CRC | The investigators hypothesize that gut microbiome composition and the four bacterial gene markers (M3) show dynamic changes after endoscopic resection of advanced neoplasia, some key bacteria are associated with restoration of gut microbiome after endoscopic resection. | No intervention | Observational | Recruiting | NCT05381792 |
| Study on the Impact of a Modified Mediterranean Diet, in Patients With CRC Undergoing Active Medical Oncology Treatment | This study evaluates the impact of the Modified Mediterranean Diet on gut microbiota diversity in metastatic CRC patients undergoing chemotherapy, comparing it to Western or standard diets. It aims to assess whether the diet promotes beneficial bacteria that support the intestinal barrier and to examine its effects on treatment side effects and quality of life. | Modified Mediterranean Diet | Interventional | Recruiting | NCT06794931 |
| CRC Associated Host and Microbiome Study | Recruit of healthy, precancer and CRC patients and record necessary information of demographic and also other messages. All the volunteers were asked to provide samples including stool, blood, urine and tissues. | No intervention | Observational | Recruiting | NCT03998644 |

| | | | | | |
|---|---|---|---|---|---|
| Gut Microbiome Dynamics in Metastasized or Irresectable CRC | In this study the characteristics and alterations of the gut microbiome during chemotherapy for metastasized or irresectable CRC are studied, as well as the relation between the gut microbiome and the effects of chemotherapy. | No intervention | Observational | Recruiting | NCT03941080 |
| Effects of Endocrine Disruptors on the Gut Microbiota and Assessment of Their Impact on CRC Development (PERMICA) | In France, lifestyle and environmental factors, including exposure to endocrine disruptors like parabens and phthalates, contribute to CRC risk, partly through gut microbiota dysbiosis. This study aims to explore the link between CRC, gut microbiota composition, and exposure to these disruptors. | Endocrine Disruptors | Interventional | Recruiting | NCT06809660 |
| Effects of Red Ginseng on Gastrointestinal Symptoms and Microbiota After Surgery for Gastrointestinal Cancer | Gastrointestinal cancer surgery often leads to symptoms like weight loss and digestive issues, likely linked to gut microbiota changes. This study evaluates whether red ginseng, with its prebiotic effects, can improve gut microbiota, gastrointestinal symptoms, and nutritional status after surgery. | Diet (Red Ginseng) | Interventional | Completed | NCT06561516 |
| Gut Microbiome Modification Through Dietary Intervention in Patients With CRC: Response to Surgery | This open-label, randomized trial at Virgen de la Arrixaca University Hospital (Spain) will enroll CRC patients scheduled for surgery. Participants will be randomized 1:1 to receive either standard nutritional guidance or a high-fibre, PUFA-rich diet (≥30g fibre and ≥3g PUFAs/day) for at least four weeks preoperatively. | High-fibre diet rich in PUFA and Standard diet | Interventional | Unknown | NCT04869956 |

| | | | | | |
|---|---|---|---|---|---|
| CARE-CRC: Microbiome Insights and Correlations for Risk and Outcomes in CRC | Lifestyle and environmental factors impact the gut microbiome and may influence cancer development. This study aims to develop a non-invasive, microbiome-based tool to enhance early CRC detection and personalized treatment. | No intervention | Observational | Not yetRecruiting | NCT06734156 |
| Mediterranean Diet and Weight Loss: Targeting the Bile Acid/Gut Microbiome Axis to Reduce CRC | This RCT in 232 obese African American adults (45–75) evaluates the effects of a Mediterranean diet, with or without calorie restriction, on bile acids, gut microbiota, and CRC-related biomarkers over 6 months. Outcomes include microbiota function, bile acid profiles, and intestinal gene expression. | Mediterranean Diet | Interventional | Recruiting | NCT04753359 |
| Impact of Dietary Intervention on Inflammation and Microbiome Composition Post-Colonoscopy | This study examines how a modified Mediterranean diet influences the gut microbiome, inflammation, and metabolism post-colonoscopy. Using stool metagenomics, serum immune profiling, and metabolomics, researchers aim to understand how diet shapes microbiome composition, immune response, and metabolic function. | Modified Plant-Based Mediterranean Diet | Interventional | Recruiting | NCT06603519 |
| The Role of Gut Microbiome and Chronic Inflammation in Young-onset CRC | This study aims to investigate the role of gut microbiome pattern and inflammation marker NF-κB in young-onset CRC | No intervention | Observational | Unknown | NCT04011969 |

| | | | | | |
|---|---|---|---|---|---|
| Calcium: Magnesium Balance, Microbiota, and Necroptosis and Inflammation | To explore the gut microbiota's role in this association, a double-blind 2×2 factorial RCT (NCT01105169) will assess whether optimizing the Ca: Mg ratio to 2.3 alters microbial abundance related to TRPM7 genotype and metachronous polyp risk across stool, swab, and tissue samples. | Dietary supplement: Magnesium glycinate and Placebo | Interventional | Completed | NCT04229992 |
| The Beans to Enrich the Gut Microbiome vs. Obesity's Negative Effects (BE GONE) Trial | The study seeks to evaluate whether consuming canned, pre-cooked beans can enhance gut microbiota and help lower cancer risk associated with obesity. A total of up to 80 participants will be recruited at MD Anderson. | Regular Diet and Navy Beans (Canned) | Interventional | Active not Recruiting | NCT02843425 |
| Ginger and Gut Microbiome (GINGER) | Estimate the impact of a 6-week daily intake of 2000 mg of ginger extract on the composition of the gut microbiome using a randomized placebo-controlled double-blinded design, i.e. examine the change of microbiome over time within and between the subjects. | Dietary supplement: Ginger extract and Placebo | Interventional | Completed | NCT03268655 |
| Dietary Supplement on the Intestinal Microbiota in Patients with Colon Cancer | Effect of a dietary supplement with antioxidant and anti-inflammatory properties on the intestinal microbiota in patients with colon cancer. Randomized placebo controlled clinical trial. Ter atrophic study | DCOOP Product, Hydroxytyrosol extract and Indukern product, Curcumin and selenium extract | Interventional | Completed | NCT05472753 |

| | | | | | |
|---|---|---|---|---|---|
| Vitamin D Intervention and Associated Changes in the Gut Microbiome and Vitamin D Levels in Healthy Adults | This study aims to assess how vitamin D supplementation affects the human gut microbiome, accounting for dietary intake. Findings may clarify the role of vitamin D and diet in shaping microbiome composition and early-onset CRC risk, potentially informing future preventive strategies. | Vitamin D intervention and Placebo intervention | Interventional | Unknown | NCT05387876 |
| Effect of the Nutraceutical "MICODIGEST 2.0" on the Complications After Surgery for CRC | Fungal extracts may help modulate microbiota and reduce inflammation. This double-blind randomized trial evaluates the effect of the fungal-based nutraceutical MICODIGEST 2.0 on post-surgical complications in CRC patients. | Dietary supplement: MICODIGEST 2.0 supplement and Placebo supplement | Interventional | Unknown | NCT04821258 |
| Study to Assess Colonic Microbiota Changes in Response to Energy Drink Consumption | This study will investigate whether short-term daily energy drink consumption results in an increase in hydrogen sulfide-producing bacteria in adults 18-40 years old. | Dietary supplement: Energy drink | Interventional | Completed | NCT06137248 |
| COLON-IM : Microbiota and Immune Infiltrate in Normal, Dysplastic and Neoplastic Colorectal Tissue | The primary objective of COLON-IM is to describe colorectal tissue microenvironment (neutrophils infiltrate) of patients with benign or malignant colorectal lesion. | No intervention | Observational | Recruiting | NCT03841799 |

| | | | | | |
|---|---|---|---|---|---|
| Bile Acids and Microbiome in Early Colorectal Carcinogenesis | This study investigates whether changes in gut microbiota and bile acid composition contribute to the progression from adenoma to CRC. Findings may identify microbial or bile acid markers for early CRC detection. | No intervention | Observational | Recruiting | NCT06502704 |
| Microbiome Test for the Detection of Colorectal Polyps and Cancer | This study aims to evaluate the sensitivity, specificity, and accuracy of the Metabiomics colon polyp and CRC assay for the non-invasive detection of colorectal polyps and cancer. | No intervention | Observational | Completed | NCT02141945 |
| Obesity, Iron Regulation and CRC Risk | This study will conduct a crossover feeding trial comparing three diets—high-iron typical American, low-iron typical American, and high-iron Mediterranean—to assess their effects on gut microbiota and inflammation. | High heme iron diet, Low iron diet and Plant-based high non-heme iron diet | Interventional | Completed | NCT03548948 |
| *Fusobacterium Nucleatum* at CRC Sites | This study investigates whether the oral cavity serves as a reservoir for *Fusobacterium nucleatum* in CRC patients. It will assess the relationship between oral, gut, and tumour colonization by *F. nucleatum*, along with dietary and microbiome factors. | Other: biopsy | Interventional | Recruiting | NCT05945082 |

| | | | | | |
|---|---|---|---|---|---|
| The Impact of Palm Date Intake on Colon Health Biomarkers | This study investigates the prebiotic effects of date fruit in healthy individuals using a 21-day crossover design with a 14-day washout. Faecal and blood samples will be analyzed to evaluate metabolic responses, microbiota shifts, and chronic disease biomarkers. | Date fruit - Ajwa variety and Maltodextrin/Dextrose | Interventional | Completed | NCT02288611 |
| Investigation of the Role of the Microbiome in the Pathogenesis of Colorectal Adenoma and Carcinoma | This study aims to elucidate host–microbiome interactions that drive adenoma formation and CRC progression. Saliva, stool, and colon biopsy samples will be collected from patients alongside dietary, lifestyle, and medical history data. Host and microbial genomes and transcriptomes will be analysed in parallel. | No intervention | Observational | Completed | NCT02947607 |
| Mesorectal Microbiome as a Prognostic Factor in Patients with Rectal Cancer | Gut microbiota imbalance is linked to various diseases, including CRC. This study aims to identify microbial profiles within the mesorectum that may predict poor outcomes, as the mesorectal microbiome remains largely unexplored. | No intervention | Observational | Recruiting | NCT04804956 |
| Microbiome and Rectal Cancer | The purpose of our study is to determine if an association exists between the microbiome of those with rectal adenocarcinoma who are complete pathologic responders and those who have a partial or no response to neoadjuvant therapy. | Neoadjuvant therapy | Interventional | Active but not Recruiting | NCT04223102 |

| | | | | | |
|---|---|---|---|---|---|
| Pilot Randomized Evaluation of Butyrate Irrigation Before Ileostomy Closure on the Colonic Mucosa in Rectal Cancer Patients | This study evaluates the impact of butyrate irrigation before ileostomy closure on colonic mucosa in rectal cancer patients. Forty-five patients will be randomized to receive butyrate, saline, or no irrigation via the efferent limb. Postoperative outcomes, microbiota composition, and bowel function will be assessed after ileostomy reversal. | Drug: Irrigations trough the efferent limb of loop ileostomy | Interventional | Completed | NCT04795180 |
| Evaluation of the Diagnostic Potential of AI-assisted Faecal Microbiome Testing for Colon Cancer | This clinical trial aims to assess whether AI-assisted faecal microbiome testing is a reliable screening tool for colon cancer. Participants will provide stool samples for next-generation sequencing. Results will be compared with colonoscopy findings to evaluate diagnostic accuracy. | AI-assisted Faecal Microbiome Testing | Interventional | Unknown | NCT05795725 |
| Fibre to Reduce Colon Cancer in Alaska Native People | A 4-week randomized trial tests whether soluble fibre supplementation can reduce colon cancer risk in Alaska Native people by increasing butyrate-producing gut microbes and lowering cancer-related biomarkers. | 70g of digestible starch and Resistant starch | Interventional | Completed | NCT03028831 |
| Meat-based Versus Pesco-vegetarian Diet and CRC | This study examines how diet-driven changes in the gut microbiome influence CRC risk. Volunteers and CRC-prone rats will follow meat-based, antioxidant-supplemented, or pesco-vegetarian diets. Microbiome, metabolome, and CRC biomarkers will be analyzed, including via faecal transplants, to clarify the microbiome's role in diet-related CRC risk. | Meat-based diet; Meat-based diet supplemented with alpha-tocopherol (MBD-T); Pesco-vegetarian diet | Interventional | Completed | NCT03416777 |

**Abbreviations:** CRC: Colorectal Cancer; TCM: Traditional Chinese Medicine; MAC: Microbiota-Accessible Carbohydrates; AI: Artificial Intelligence; NCT: National Clinical

Trial; RCT: Randomized Controlled Trial.

**Table S2.** Microbiota-directed (prebiotic, probiotic, synbiotic, postbiotic) intervention for CRC in clinical trial.

| Study Title | Brief Summary | Interventions | Study Type | Study Status | NCT Number |
|---|---|---|---|---|---|
| **Prebiotic-based intervention** | | | | | |
| Prebiotics in Rectal Cancer | While the gut microbiome is known to influence tumour development and immune response, it remains unclear whether microbiome-targeted therapies can improve CRC outcomes. | Soluble Corn Fibre and Maltodextrin | Interventional | Unknown | NCT05516641 |
| Prebiotic Effect of Eicosapentaenoic Acid Treatment for CRC Liver Metastases | This study, linked to the EMT2 trial (NCT03428477), investigates how the omega-3 fatty acid EPA may prevent cancer recurrence after liver surgery for colorectal metastases. By analyzing stool, urine, blood, and tumour samples, it aims to uncover microbiome and immune mechanisms behind EPA's effects and identify patients most likely to benefit, supporting personalized treatment approaches. | Drug: Icosapent Ethyl Oral Capsule | Interventional | Completed | NCT04682665 |
| **Probiotic-based intervention** | | | | | |

| | | | | | |
|---|---|---|---|---|---|
| Impact of Probiotics on the Intestinal Microbiota | This study aims to evaluate the effects of probiotic administration (*Saccharomyces boulardii*) in patients undergoing colorectal resection compared to standard care. Outcomes include: (1) modulation of intestinal microbiota and (2) postoperative outcomes. | Dietary supplement: *Saccharomyces boulardii* | Interventional | Completed | NCT01609660 |
| Impact of Probiotics in Modulation of Intestinal Microbiota | The investigators would study about impact of the administration of probiotics in the intestinal mucosa of patients undergoing resection colic, by evaluating cytokine profile by quantitative real time PCR. | Dietary supplement: *Saccharomyces boulardii* | Interventional | Completed | NCT01895530 |
| Microbiota Implementation to Reduce Anastomotic Colorectal Leaks | Aim of this study is to implement the intestinal microbiota by perioperative administration of probiotics, oral antibiotics and low volume mechanical preparation. | Probiotics, oral antibiotics and mechanical preparation | Interventional | Completed | NCT05164887 |
| Oral *Lactobacillus Rhamnosus* TCELL-1 and CRC | This study evaluates the effects of *Lactobacillus rhamnosus* TCELL-1—isolated from healthy Taiwanese—on stage III CRC patients undergoing adjuvant chemotherapy. | Dietary supplement: Oral *Lactobacillus rhamnosus* TCELL-1 | Interventional | Unknown | NCT05570942 |

| | | | | | |
|---|---|---|---|---|---|
| Akkermansia Probiotics Plus Anti-PD-1 Monoclonal Antibody in MSS/pMMR Advanced CRC | The investigators propose to conduct a single-center, single-arm, Phase I clinical study to explore the safety and feasibility of Akkermansia probiotics combined with anti-PD-1 monoclonal antibody in patients with MSS/pMMR advanced CRC, as well as its impact on gut microbiota and the immune microenvironment. | Akkermansia Probiotics; PD-1 Inhibitor | Interventional | Active not Recruiting | NCT06865521 |
| Using Probiotics to Reactivate Tumour Suppressor Genes in Colon Cancer | This study aims to determine whether probiotic supplementation can positively influence colon cancer-associated microbiota and epigenetic changes. Participants will receive two daily ProBion Clinica tablets containing *Bifidobacterium lactis* Bl-04, *Lactobacillus acidophilus* NCFM, and inulin. | Dietary supplement: ProBion Clinica | Interventional | Completed | NCT03072641 |
| Probiotics In CRC Patients | This double-blind, randomized trial assesses bacterial colonization at surgery (Day 0), impacts on gut microbiota and immune response, and explores dose-dependent colonization of *Lactobacillus acidophilus La1* and its influence on microbial and immunological outcomes. | Procedure: Probiotics (La1, BB536) and placebo | Interventional | Completed | NCT00936572 |
| Probiotics Combined With Standard Chemotherapy Plus Targeted Therapy in Patients With Metastatic CRC | This study evaluates whether adding Bifico, a probiotic with proven gut and anti-cancer benefits, enhances the efficacy of standard chemotherapy and targeted therapy in metastatic CRC. It also explores links between gut microbiota, immune function, and patient prognosis to identify predictive biomarkers. | Chemotherapy plus targeted therapy | Interventional | Not yet Recruiting | NCT04131803 |

| | | | | | |
|---|---|---|---|---|---|
| An Evaluation of Probiotic in the Clinical Course of Patients With CRC | This study investigates whether probiotic functional foods can reduce inflammation and improve symptoms in CRC patients by modulating the gut microbiome. | Dietary supplement: Probiotic | Interventional | Completed | NCT03782428 |
| **Synbiotic-based intervention** | | | | | |
| Effect of the Use of Symbiotics in Patients With Colon Cancer | This study evaluates whether perioperative symbiotic supplementation can improve gut function and clinical outcomes in colon cancer patients undergoing colorectal surgery. | Symbyotic and Control Group | Interventional | Unknown | NCT04874883 |
| Effect of Synbiotic Supplementation on the Prevention of Mucositis in Cancer Patients Undergoing Chemotherapy | This randomized clinical trial evaluates whether pre-chemotherapy synbiotic supplementation can reduce mucositis and diarrhoea in CRC patients treated with capecitabine, aiming to improve gut health and quality of life. | Dietary supplement: Symbiotic | Interventional | Recruiting | NCT06576986 |
| Action of Synbiotic on Irradiated GI Mucosa in Rectal Cancer Treatment | The aim of this study is to investigate how bacteria and fibre interact with the epithelial cells of the gastrointestinal mucosa to reduce inflammation and to diminish tissue damage caused by radiation therapy. | Oat bran and blueberry husks | Interventional | Completed | NCT03420443 |
| **Postbiotic-based intervention** | | | | | |

| | | | | | |
|---|---|---|---|---|---|
| Postbiotics for Mitigation of Postoperative Dysbiosis in Colon Cancer Surgery | This study assesses the efficacy of postbiotic supplements in reducing gut dysbiosis after colon cancer surgery by measuring changes in faecal Shannon Diversity Index (SDI) from baseline to postoperative timepoints at 2 weeks post-surgery. | Dietary supplement: PoZibio and Inert placebo | Interventional | Not Yet Recruiting | NCT07050485 |

**Table S3.** Drug-mediated modulation of Gut-microbiome of CRC in clinical trial.

| **Study Title** | **Brief Summary** | **Interventions** | **Study Type** | **Study Status** | **NCT Number** |
|---|---|---|---|---|---|
| Coffee and Metabolites Modulating the Gut Microbiome in CRC | This study is assessing the effects of 6-g daily use of freeze-dried instant coffee on liver fat and fibrosis and the gut microbiome and metabolome in patients who have completed routine treatment (including surgery, chemotherapy and radiotherapy) for stage I-III CRC. | Drug: Nestlé NESCAFÉ® TASTER'S CHOICE® House Blend capsule and Placebo | Interventional | Recruiting | NCT05692024 |
| Omega 3 Fatty Acids in CRC Prevention in Patients With Lynch Syndrome | This is a pilot study aimed at assessing the effects of moderate dose omega-3-acid ethyl esters capsules (generic Lovaza) on molecular, and intestinal microbiota changes in participants at high risk for CRC. The study will be a single arm, open label study. | Drug: Omega-3 fatty acid ethyl esters (2 gram) | Interventional | Unknown | NCT03831698 |

| | | | | | |
|---|---|---|---|---|---|
| Gut Microbiome and Its Immune Modulation in Locally Advanced Rectal Cancer | To evaluate the efficacy and safety of TNT with GEN-001 (Lactococcus lactis) and identify predictive biomarkers for pathologic response in patients with locally advanced rectal cancer (LARC). | Drug: TNT with GEN-001 (Lactococcus lactis) | Interventional | Unknown | NCT05079503 |
| OMega-3 Fatty Acid for the Immune Modulation of CRC | This trial evaluates whether daily 4g marine omega-3 (VASCEPA) alters the tumour immune environment and gut microbiome in CRC patients before surgery, using a double-blind, placebo-controlled design. | Drug: AMR101 (VASCEPA, icosapent ethyl) | Interventional | Withdrawn | NCT03661047 |
| Study of Berberine Hydrochloride in Prevention of Colorectal Adenomas Recurrence | In recent years, anticancer activity of berberine hydrochloride have been explored. The aim of this study is to investigate the effect of berberine hydrochloride on the recurrence of colorectal adenomas. | Drug: Berberine hydrochloride | Interventional | Completed | NCT02226185 |
